# Homogeneous Copper(I) Electrocatalyzed Degradation of Ultra‐Short and Long‐Chain Perfluoroalkyl Substances

**DOI:** 10.1002/cssc.202502358

**Published:** 2026-04-18

**Authors:** Soumalya Sinha, Ashwin Chaturvedi, Nabin Pandey, Julien A. Panetier, Jianbing “Jimmy” Jiang

**Affiliations:** ^1^ Department of Chemistry University of Cincinnati Cincinnati Ohio USA; ^2^ Department of Chemistry State University of New York Binghamton New York USA

**Keywords:** C—F bond activation, electrocatalysis, perfluoroalkyl substance degradation, short‐/long chain perfluoroalkyl substance

## Abstract

While the defluorination of environmentally persistent poly‐ and perfluoroalkyl substances (PFASs) requires the development of energy‐efficient methodologies for complete mineralization, electrochemical C—F bond activation remains a fundamental research challenge. Although C—F activation chemistry is commonly studied using fluoromethylarenes as substrates, the stronger C—F bonds in short‐chain perfluoroalkyl carboxylic acids (PFCAs), fluorinated aliphatic alcohols, and fluorotelomers are yet to be explored. Herein, we report a molecular copper(I) complex that activates the C—F bonds in trifluoroacetic acid (**TFA**), 2,2,2‐trifluoroethanol (**TFE**), 2,2‐difluoroethanol (**DFE**), and 2‐fluoroethanol (**MFE**) when a constant reductive current of 6 mA is applied. 99% free fluoride was recovered from **MFE** after 12 h of electrolysis, which is comparatively higher than that of **TFE** (∼15%), **DFE** (19.2%), or **TFA** (∼14%), and is attributable to the strengths of the C—F bonds in these molecules. We also defluorinated four selected PFCAs bearing short‐chain C—F backbones and terminal trifluoromethyl groups, which are common end products formed by the degradation of toxic perfluoroalkyl substances. In addition, we investigated the defluorination and degradation of two fluorotelomers under ambient conditions. Density functional theory calculations revealed a correlation between the %fluoride recovery and the C—F bond dissociation energy of each substrate. Accordingly, we report a rare example of a molecular electrocatalytic system that activates the strong C—F bonds in fluoromethylalkyls, including short‐chain PFASs, which are typical end products of common PFAS degradation processes.

## Graphical abstract

Molecular copper(I) electrocatalyst promoted ultra‐short and long‐chain perfluoroalkyl substance (PFAS) degradation under ambient homogeneous electrochemical conditions. Substrate scopes also include fluoroalkyl alcohols and trifluoroacetic acid. Controlled‐current electrolysis studies in the presence of the copper(I) catalyst showed the defluorination of resilient C–F bonds under ambient conditions.

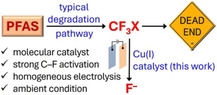

## 1 Introduction

Activating the C—F bonds in poly‐ and perfluoroalkyl carboxylic acids (PFCAs), which are common groundwater contaminants, has been a key challenge for their mineralization [1, 2, 3, 4]. Although the C—F bond is highly polarized, the net electrostatic interaction between the polarized C^δ+^ and F^δ–^ endows the bond with significant stability and a bond dissociation energy (BDE) greater than 105 kcal/mol [5, 6, 7, 8, 9, 10]. Therefore, activating a C—F bond requires overcoming a high activation energy barrier, for which determining mild operating conditions is challenging. Many methods, including the use of high doses of chemical oxidants/reductants [11], ultraviolet exposure [5, 12, 13, 14], and metalloenzymes [15], as well as mechanochemical [16, 17, 18], photochemical [19, 20, 21, 22], and electrochemical [23, 24, 25, 26, 27] strategies, are promising for cleaving typical C—F bonds in PFCAs or fluorinated organic compounds [28]. However, common drawbacks include harsh reaction conditions and the use of toxic chemicals. Although these strategies effectively break the C—F bonds in the ‐CF_2_‐ backbone of a PFCA, the final degraded products are typically short‐chain PFCAs with trifluoromethyl (CF_3_) terminal groups whose further defluorination remains challenging owing to the comparatively strong C—F bonds in the CF_3_ moiety (117 kcal/mol) [5, 29, 30]. In addition, activating C—F bonds in shorter‐chain PFCAs is more challenging than those in longer‐chain ones with more than six carbon atoms [5, 13]. In this study, we focused on electrochemically defluorinating shorter PFCA chains and organofluorine compounds bearing CF_3_ groups under ambient conditions.

Reported defluorination strategies typically focus on fluoroarenes, trifluoromethylarenes, α‐trifluoromethylalkenes, difluoroalkenes, and trifluoromethylketones [31, 32, 33, 34, 35], while studies involving saturated fluoro‐aliphatic acids or alcohols have rarely been reported. Chemically treating PFCAs and fluorotelomers in aprotic solvents with NaOH at 120°C for 24 h leads to the formation of free fluoride ions (F^–^) along with trifluoroacetate (CF_3_CO_2_^–^) as a byproduct [29, 36]. However, CF_3_CO_2_^–^ was unable to be further defluorinated under identical chemical conditions, and molecular electrocatalysts that activate the C—F bonds in the CF_3_ group have not been reported.

**FIGURE 1:**
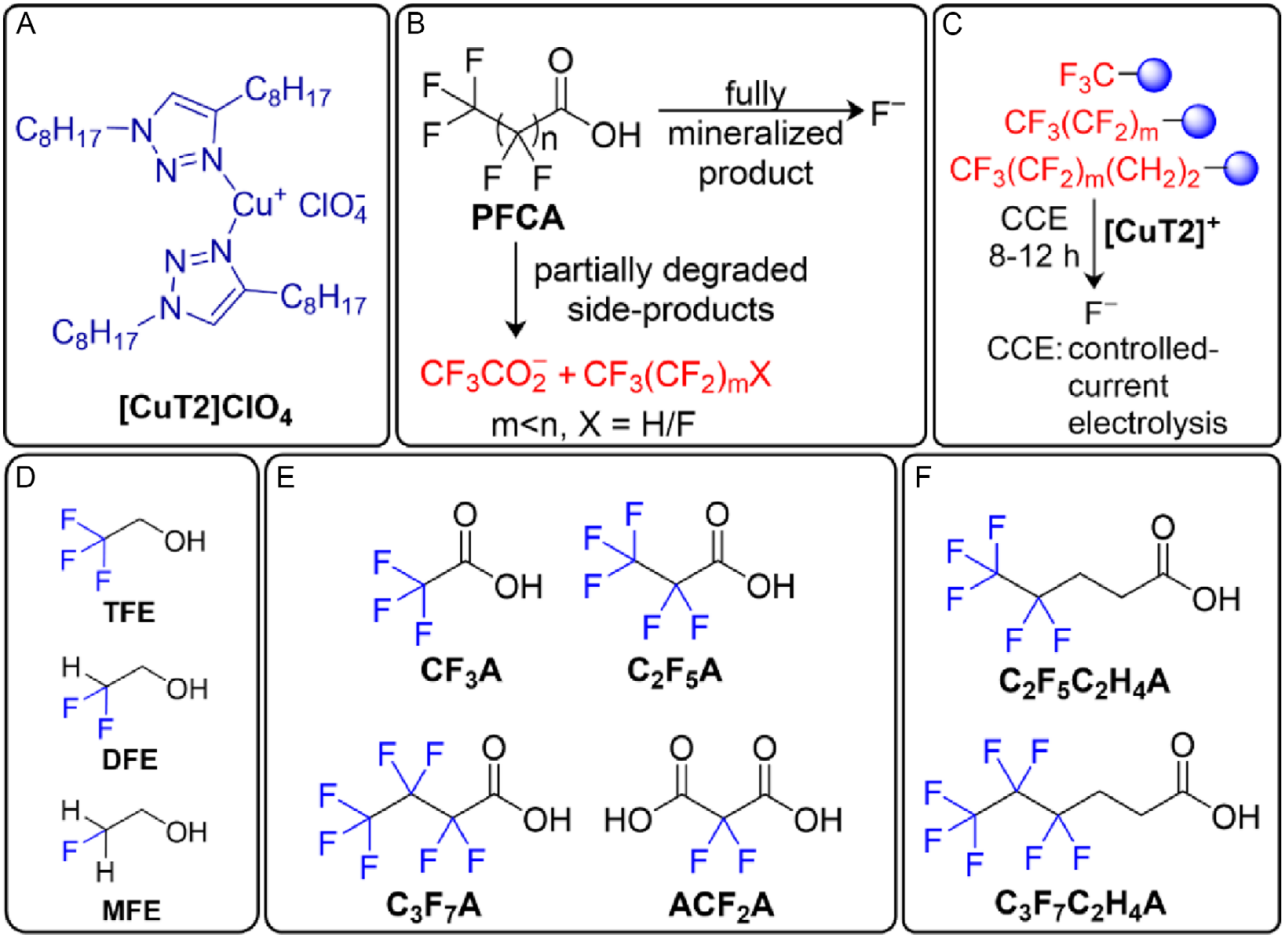
(A) The **[CuT2]ClO**_**4**_ molecular Cu(I) catalyst. (B) Fully mineralized and partially degraded products produced by PFCA decomposition processes. (C) Electrochemical activation of the C–F bonds in the CF_3_‐group‐containing systems studied in this work. The (D) three perfluoroalkyl alcohols, (E) four PFCAs, and (F) two fluorotelomers investigated in this study.

We recently reported **[CuT2]ClO**_**4**_, a molecular Cu(I) electrocatalyst bearing triazole‐based ligands (Figure 1A) that defluorinates perfluorooctanoic acid (PFOA) at a rate of 99% within 4 h of controlled‐current electrolysis (CCE) under homogeneous ambient conditions [30]. However, CF_3_‐containing byproducts, such as CF_3_CO_2_^–^, CF_3_H, and CF_4_, were detected during analysis of the postelectrolysis solution and headspaces of the electrochemical cells (Figure 1B), which suggests that the terminal CF_3_ group in PFOA mainly remained intact [30]. Inspired by our previous work, we now report the electrochemical defluorination performance of **[CuT2]ClO**_**4**_ toward six CF_3_‐containing substrates under homogeneous CCE conditions (Figure 1C–F), namely 2,2,2‐trifluoroethanol (**TFE**), trifluoroacetic acid (**TFA**, **CF**_**3**_ **A**), pentafluoropropionic acid (**C**_**2**_ **F**_**5**_ **A**), perfluorobutanoic acid (**C**_**3**_ **F**_**7**_ **A**), 4,4,5,5,5‐pentafluoropentanoic acid (**C**_**2**_ **F**_**5**_ **C**_**2**_ **H**_**4**_ **A**), and 4,4,5,5,6,6,6‐heptafluorohexanoic acid (**C**_**3**_ **F**_**7**_ **C**_**2**_ **H**_**4**_ **A**), where **A** refers to the “carboxylic acid” (CO_2_H) moiety. We also studied analogs devoid of terminal CF_3_ groups (Figure 1D,E), including 2,2‐difluoroethanol (**DFE**), 2‐fluoroethanol (**MFE**), and difluoropropanedioic acid (**ACF**_**2**_ **A**), to compare the rates of **[CuT2]ClO**_**4**_‐promoted defluorination. **[CuT2]ClO**_**4**_ effectively activated the C—F bonds in all of the abovementioned fluoroalkyl substances when a reductive current (–1 or –6 mA) was constantly applied over 8–12 h at room temperature. Free F^–^ was recovered from post‐CCE solutions and quantified by ion chromatography (IC). The %fluoride recovery was found to depend on the strength of the C—F bond. This study reveals a rare molecular electrocatalyst capable of activating C—F bonds in both CF_3_‐ and CF_2_‐containing alkyl fluorides.

## 2 Results and Discussion

We synthesized and characterized **[CuT2]ClO**_**4**_ according to our previously reported method [30]. All CCE experiments were performed in a membrane‐separated H‐cell at room temperature under N_2_. A nonaqueous electrolyte was prepared by adding 0.1 M tetrabutylammonium perchlorate (TBAClO_4_) to MeCN as the solvent, which we refer to as the “MeCN electrolyte”. The reported potentials were calibrated against the ferrocene–ferrocenium redox couple (Fc/Fc^+^), and reductively applied currents are indicated with negative signs. Further experimental details are provided in the Experimental Section below.

### 2.1 Defluorinating Fluoroalkyl Alcohols

**FIGURE 2:**
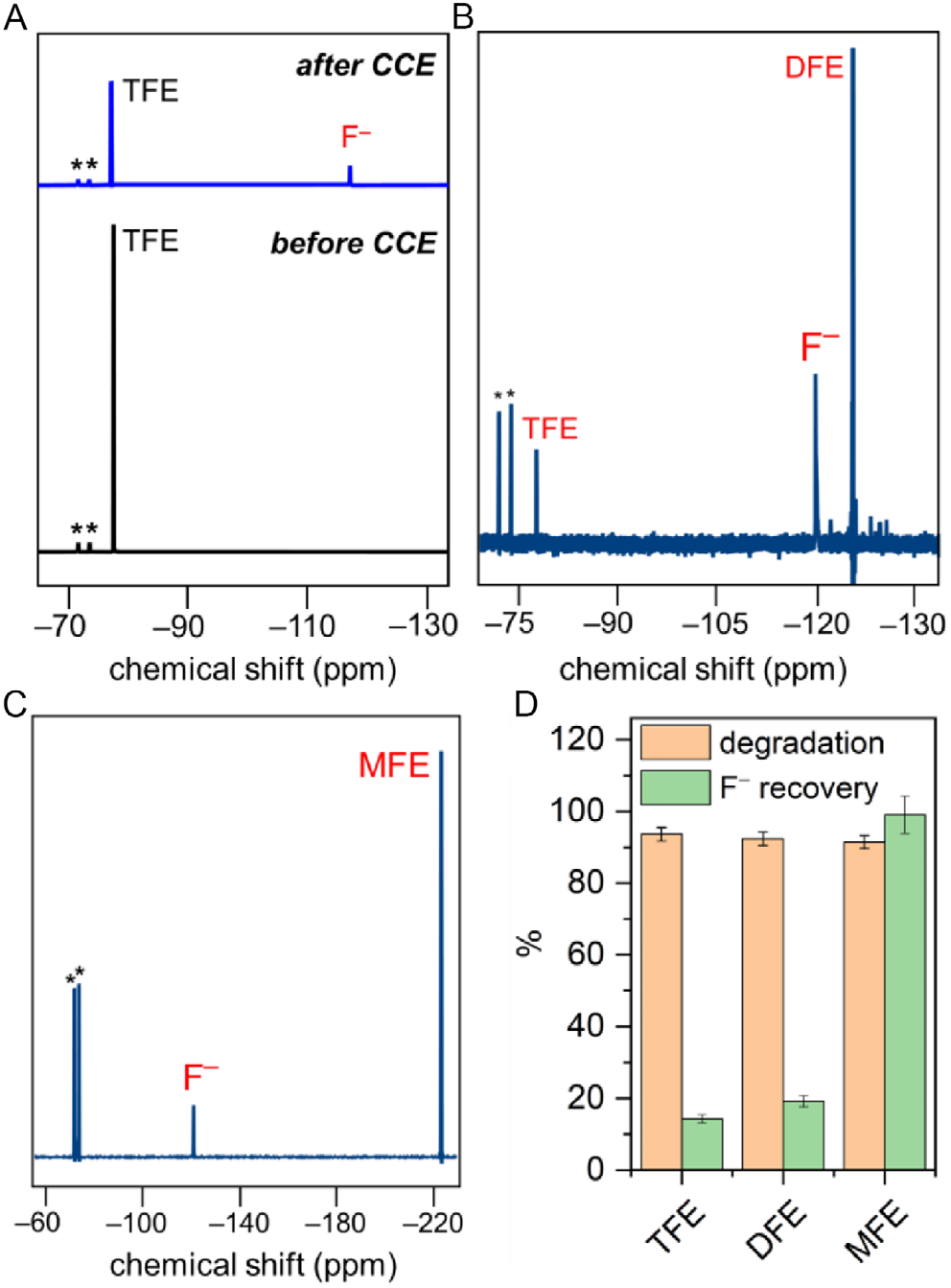
(A) Comparative ^19^F NMR spectra recorded for a catholyte solution containing 0.86 M **TFE** and 1 mM **[CuT2]ClO**_**4**_ before and after controlled‐current electrolysis (CCE) at –6 mA for 12 h. ^19^F NMR spectra of post‐CCE catholyte solutions of degraded (B) **DFE** and (C) **MFE**. (D) Degradation and F^–^ recovery percentages for **TFE**, **DFE**, and **MFE** after CCE. TBAPF_6_ was used as the internal standard, and its NMR peaks are marked with asterisks (*). ^19^F NMR peak integration values are listed in Table S1 in the Supporting Information.

We applied a current of –6 mA for 12 h under CCE conditions to 1 mM **[CuT2]ClO**_**4**_ and 0.86 M **TFE** in the MeCN electrolyte to defluorinate the CF_3_ group in **TFE**. The potential at the working electrode shifted from –2.1 to –2.9 V versus Fc/Fc^+^ during CCE (Figure S1), which is comparatively more positive than that of the bare electrode in the absence of **[CuT2]ClO**_**4**_ under identical electrochemical conditions (Figure S2). The headspace of the cathodic compartment of the electrochemical H‐cell was analyzed using gas chromatography (GC), which, as expected, revealed the formation of H_2_ during CCE at potentials more negative than –2.1 V versus Fc/Fc^+^ in the presence of **TFE** [37]. The ^19^F nuclear magnetic resonance (NMR) spectrum of the post‐CCE catholyte solution revealed a new peak at –117 ppm (Figure 2A) that was not present in the spectrum of the pre‐CCE electrolyte. However, this peak disappeared when the post‐CCE catholyte was exposed to the air for more than 1 h, while a new broad peak appeared at –151 ppm (Figure S3). We recorded a ^19^F NMR spectrum for commercially purchased tetrabutylammonium fluoride trihydrate (TBAF•3H_2_O) in CD_3_CN to provide an understanding of the observed NMR behavior; this sample exhibited a signal at –117 ppm for F^–^ and an additional peak at –151 ppm that corresponds to hydrated fluoride, HF_2_^–^ (Figure S3) [38]. Both NMR peaks are broad, which is possibly ascribable to spontaneous interchange between F^–^ and HF_2_^–^ in the pure TBAF•3H_2_O solution. Accordingly, we conclude that the peak at –117 ppm in the ^19^F NMR spectrum of the post‐CCE catholyte is due to free F^–^, which transformed into HF_2_^–^ upon exposure to moisture, which indicates the C—F bonds in **TFE** were activated by the **[CuT2]ClO**_**4**_ in the MeCN electrolyte. The post‐CCE solution following **TFE** defluorination for 12 h at –6 mA in the presence of the Cu(I) catalyst was also analyzed by IC, which revealed 14.3% F^–^ in the post‐CCE catholyte (Figure S4). We investigated the stability of the supporting electrolyte (TBAClO_4_) and solvent (MeCN) under the applied CCE conditions. ^1^H NMR data collected before and after 12 h of CCE using the 0.1 M TBAClO_4_ in MeCN solution in the presence of **[CuT2]ClO**_**4**_ did not show any significant changes or additional ^1^H NMR signals after the electrolysis (Figure S5), suggesting that the electrolyte molecules and solvent system are stable under the applied CCE conditions.

We also investigated the reactivity of F^–^ formed during the activation of the C—F bonds in **TFE** under CCE conditions using **[CuT2]ClO**_**4**_, which further confirmed that free F^–^ is generated in the CCE experiment. In theory, F^–^ is the only halide capable of reducing Ag(I) to Ag(0) to form a silver mirror on the inner surface of a glass vial [38]. This reactivity was tested by collecting 3 mL of the post‐CCE solution immediately upon completion of the abovementioned 12 h CCE experiment and adding 5 mg of silver triflate (AgOTf) to it. The mixture was filtered and the colorless filtrate was collected in a glass vial. The filtrate eventually turned into a metallic gray solution over time, followed by the formation of a silver mirror on the surface of the glass vial (Figure S6), which suggests that F^–^ is present in the post‐CCE solution after subjecting **[CuT2]ClO**_**4**_ and **TFE** to 12 h of CCE in the MeCN electrolyte. A control experiment in which an identical amount of AgOTf was reacted with the same amounts of **[CuT2]ClO**_**4**_ and **TFE** used in the above‐mentioned CCE experiment, but in the absence of CCE, was also performed (Figure S6). No silver mirror was formed on this occasion, confirming that F^–^ is produced from **TFE** in response to CCE in the presence of **[CuT2]ClO**_**4**_. An additional control experiment using pure TBAF•3H_2_O in the MeCN electrolyte resulted in the formation of a silver mirror upon reaction with AgOTf.

We used rinse‐test experiments to further investigate the homogeneous nature of **[CuT2]ClO**_**4**_ while activating the C—F bonds in **TFE** during CCE. The carbon paper working electrode used during electrolysis was removed from the catholyte solution at the end of the CCE procedure and gently rinsed with dry MeCN to remove any loosely bound molecules on the electrode surface, and then reimmersed in freshly prepared MeCN electrolyte with an identical amount of **TFE** but without **[CuT2]ClO**_**4**_. No signal corresponding to F^–^ was observed by either ^19^F NMR spectroscopy or IC when CCE was performed under these electrochemical conditions, from which we conclude that **[CuT2]ClO**_**4**_ or metallic copper, which may have activated the C—F bonds in **TFE** under CCE conditions, does not adsorb on the surface of the carbon electrode [30]. To test that **[CuT2]ClO**_**4**_ maintained the homogeneous condition under the electrochemical condition applied in CCE, we performed cyclic voltammetry (CV) at different scan rates in a membrane‐separated H‐cell using the identical electrode setup, as used in the CCE (Figure S7A). The peak current densities showed linearity with the square root of the scan rates (Figure S7B), suggesting a diffusion‐controlled process, as expected for a purely homogeneous system [39]. Furthermore, the bare carbon paper electrode and the same electrode immersed in 1 mM **[CuT2]ClO**_**4**_ in MeCN for 15 min, were examined by scanning electron microscopy (SEM), which revealed the absence of even trace amounts of copper on the electrode surface (Figures S8 and S9), suggesting that **[CuT2]ClO**_**4**_ does not adsorb on the electrode surface. However, a small amount of metallic Cu deposition was observed on the electrode by SEM after CCE at –6 mA for 12 h in the presence of **[CuT2]ClO**_**4**_ and **TFE** (Figure S10). Energy‐dispersive X‐ray (EDX) spectroscopy revealed that 2.5 wt% Cu was deposited on the carbon surface (Figures S11 and S12).

**[CuT2]ClO**_**4**_ was also subjected to controlled potential electrolysis (CPE) experiments in the presence of **TFE** in the MeCN electrolyte to benchmark **[CuT2]ClO**_**4**_ stability at potentials more negative than –2.0 V versus Fc/Fc^+^. CPE carried out at –2.1 V versus Fc/Fc^+^ led to lower current densities after 1 h; the magnitude of the maximum current density was observed to decrease with increasing negative potential (Figure S13). A metallic Cu deposition was visible on the surface of the electrode, which reveals that **[CuT2]ClO**_**4**_ is not stable upon prolonged exposure to fixed applied potentials lower than –2.1 V versus Fc/Fc^+^ (Figure S14). In addition, the carbon paper electrode exhibited a layer of metallic Cu by SEM following CPE at –2.1 V versus Fc/Fc^+^ (Figure S15), with EDX further revealing that 57.9 wt% Cu was deposited (Figure S16). However, no such prominent deposition of metallic Cu was observed over 12 h of CCE with **[CuT2]ClO**_**4**_ in the presence of **TFE** at an applied cathodic current of –6 mA, which indicates that **[CuT2]ClO**_**4**_ is stable under homogeneous CCE conditions, as discussed in our previous report [30].

We also investigated the byproducts formed from C—F bond‐activated **TFE** in the CCE experiment with **[CuT2]ClO**_**4**_. While we anticipated that **DFE** and **MFE** might be formed as side‐products if the –CF_3_ group in **TFE** is activated in a stepwise manner, no relatable ^19^F NMR signals were observed in the spectrum of the post‐CCE catholyte solutions after 12 h of CCE using **[CuT2]ClO**_**4**_. We searched for any ethanol signal in the ^1^H NMR spectrum of the post‐CCE solution; however, this was complicated owing to the high concentrations of supporting electrolytes. We also attempted to precipitate most of the electrolyte molecules from the post‐CCE solution by adding excess diethyl ether; however, the ^1^H NMR signals for the ether overlap with those of ethanol if present. Our efforts to detect ethanol using gas chromatography–mass spectrometry (GC–MS) were unsuccessful because ethanol elutes with MeCN within the solvent‐delay time. Unfortunately, optimizing the GC–MS solvent‐delay time to enable the detection of ethanol was unsuccessful because the ethanol signal was convoluted by the solvent peak.

In a similar manner, we examined the defluorination of **DFE** and **MFE**, both at a concentration of 0.86 M, in the presence of **[CuT2]ClO**_**4**_ under homogeneous CCE conditions at a current of –6 mA for 12 h. ^19^F NMR spectroscopy revealed the formation of free F^–^ in each case (Figure 2B,C) when the post‐CCE catholytes were analyzed. In addition, a ^19^F NMR signal for **TFE** was observed in the spectrum of the post‐CCE catholyte for the **DFE** substrate, which reveals that some F^–^ rebinds to the C–H‐activated **DFE** intermediate [40] likely formed during CCE to generate **TFE** as a side product. Although **DFE** and **MFE** exhibited similar degradation rates (>92%) to those observed for **TFE**, they showed comparatively higher defluorination rates of 19% and 99%, respectively (Figure 2D). These data indicate that **TFE** is defluorinated less than **DFE** or **MFE** owing to the stronger C—F bonds of the –CF_3_ group in **TFE**. For comparison, **TFE**, **DFE**, and **MFE** were calculated to have C—F bond strengths of 123.8, 118.5, and 112.3 kcal/mol, respectively, using computational techniques (see Experimental Section for computational details). Under the identical CCE condition, a higher %fluoride recovery from **MFE** was observed compared to that from **DFE**. Performing CCE at different concentrations of **[CuT2]ClO**_**4**_ did not influence the %fluoride recovery values (Table S2). We speculate that obtaining higher free F^–^ ions from **MFE** is because of the comparatively weaker C—F bond in **MFE** (112.3 kcal/mol) than that in **DFE** (118.5 kcal/mol).

### 2.2 Defluorinating PFCAs

**FIGURE 3:**
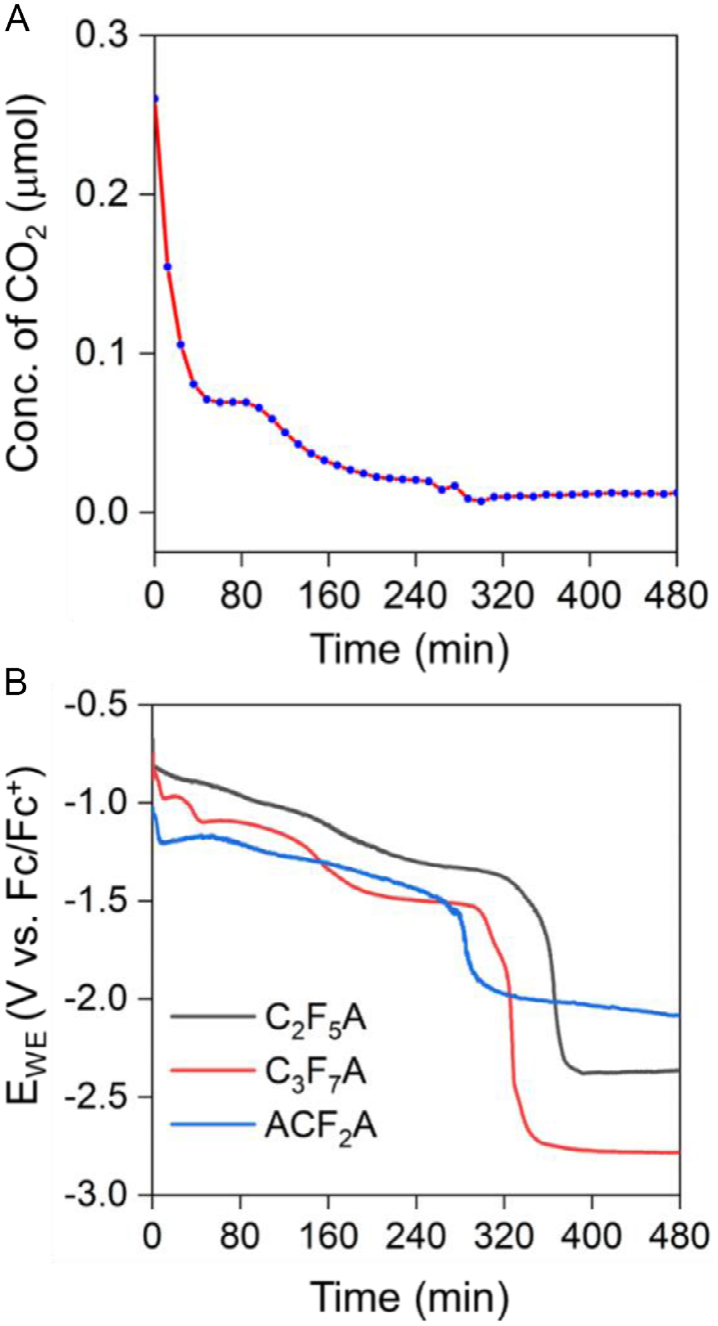
(A) CO_2_ concentrations detected in the headspace of the cathodic compartment as functions of time during CCE of **C**_**2**_ **F**_**5**_ **A** and **[CuT2]ClO**_**4**_ at –1 mA over 8 h in N_2_‐saturated MeCN as the electrolyte. (B) E_WE_ values as functions of time for **C**_**2**_ **F**_**5**_ **A**, **C**_**3**_ **F**_**7**_ **A**, and **ACF**_**2**_ **A** electrolyzed under the same conditions as those in panel (A).

We also explored activating the resilient C—F bonds in various short‐chain PFCAs (Figure 1E), including **CF**_**3**_ **A**, **C**_**2**_ **F**_**5**_ **A**, **C**_**3**_ **F**_**7**_ **A**, and **ACF**_**2**_ **A**. Catholyte solutions containing 1 mM **[CuT2]ClO**_**4**_ and 36 mM **C**_**2**_ **F**_**5**_ **A** or **C**_**3**_ **F**_**7**_ **A** were subjected to homogeneous CCE at a current of –1 mA for 8 h. The headspace of the cathode compartment of the H‐cell was analyzed by GC, with CO_2_ clearly evolved during CCE in the presence of these substrates (Figure 3A). An exponential decrease in CO_2_ formation was observed during the first 4 h of electrolysis, which plateaued to a low near‐baseline value, consistent with complete decarboxylation of **C**_**2**_ **F**_**5**_ **A** or **C**_**3**_ **F**_**7**_ **A** during the first 4 h of CCE [30]. Both **C**_**2**_ **F**_**5**_ **A** and **C**_**3**_ **F**_**7**_ **A** exhibited similar time‐dependent working electrode potentials (E_WE_) (Figure 3B) during CCE; they were also similar to those reported previously for the **[CuT2]ClO**_**4**_ **‐**electrocatalyzed decomposition of PFOA in the MeCN electrolyte under homogeneous CCE conditions [30]. We described a region in the E_WE_ vs. time profile in which E_WE_ was more positive than –2 V vs. Fc/Fc^+^, which corresponds to the decarboxylation of PFOA, followed by a subsequent E_WE_ segment in which the C—F backbone defluorinates [30]. Similarly, the quasiplateau potentials observed for **C**_**2**_ **F**_**5**_ **A** and **C**_**3**_ **F**_**7**_ **A** during the last 2 h of CCE are attributable to the defluorination of these substrates. Notably, **C**_**3**_ **F**_**7**_ **A** exhibited a more negative quasi‐plateau potential over the last 2 h of CCE than **C**_**2**_ **F**_**5**_ **A** (Figure 3B), which indicates that **C**_**3**_ **F**_**7**_ **A**, which contains an extra –CF_2_‐ group in its backbone, is more challenging to defluorinate.

**FIGURE 4:**
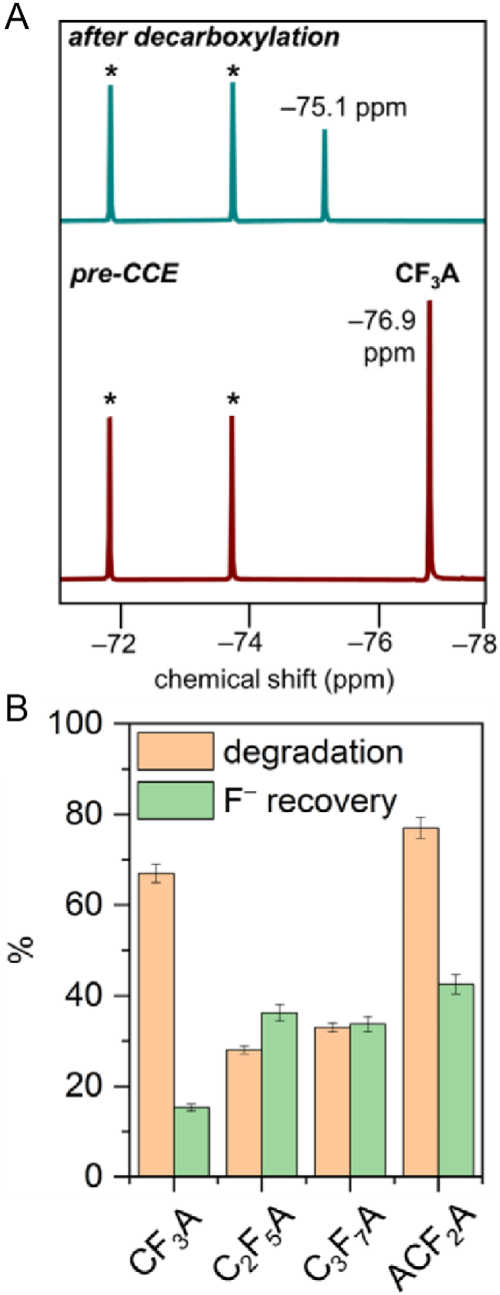
(A) Comparative ^19^F NMR spectra recorded before and after CCE at –1 mA in the presence of 36 mM **CF**_**3**_ **A** and 1 mM **[CuT2]ClO**_**4**_ in the MeCN electrolyte. (B) Degradation and defluorination rates recorded for **CF**_**3**_ **A**, **C**_**2**_ **F**_**5**_ **A**, **C**_**3**_ **F**_**7**_ **A**, and **ACF**_**2**_ **A** after 8 h of electrolysis at –1 mA in the presence of **[CuT2]ClO**_**4**_ in the MeCN electrolyte. TBAPF_6_ was used as the internal standard, and its NMR peaks are marked with asterisks (*).

We also examined the electrochemical defluorination of **CF**_**3**_ **A**, in which the CF_3_ group is the only fluoroalkyl unit in addition to the terminal carboxyl group. **CF**_**3**_ **A** (36 mM) was subjected to CCE at –1 mA in the presence of **[CuT2]ClO**_**4**_. Decarboxylation was initially observed, as discussed above for **C**_**2**_ **F**_**5**_ **A** and **C**_**3**_ **F**_**7**_ **A**, as indicated by the shift in the ^19^F NMR signal of the CF_3_ group from –76.9 to –75.1 ppm during CCE (Figure 4A). The ^19^F NMR signal at –75.1 ppm could be for the decarboxylated species of CF_3_CO_2_^–^, such as CF_3_^+^ or •CF_3_ radical, but the detailed identity of those species was challenging to elucidate. **CF**_**3**_ **A** was degraded by 67.6% and 15.2% of free F^–^ ions were recovered after CCE at –1 mA (Figure 4B), which are similar values to those obtained for **TFE** under identical electrochemical conditions. The low defluorination percentages recorded for both **CF**_**3**_ **A** and **TFE** are ascribable to the strong C—F bonds in the CF_3_ group. Interestingly, increasing the CCE current from –1 to –6 mA did not increase the defluorination rates of these compounds. Figure S17 shows ^19^F NMR spectra of the post‐CCE catholyte solutions of the **CF**_**3**_ **A** substrate subjected to –6 mA. We also performed CCE at –6 mA for PFOA by using **[CuT2]ClO**_**4**_, which did not show the formation of the CF_3_CO_2_^–^ ion in the ^19^F NMR that we observed in our previous study under –3 mA of applied current [30]. These results suggest that a small amount of CF_3_CO_2_^–^ formed during the PFOA degradation can be further defluorinated in the presence of **[CuT2]ClO**_**4**_ by increasing the amount of applied reductive current in CCE.

The ^19^F NMR data presented in Figures S18 and S19 yielded degradation rates of 28% and 33% for **C**_**2**_ **F**_**5**_ **A** and **C**_**3**_ **F**_**7**_ **A**, respectively, following CCE in the presence of **[CuT2]ClO**_**4**_ (Figure 4B). These results are consistent with the theoretical prediction that PFCAs with longer chains degrade more easily [5]. Moreover, PFCAs with more than five –CF_2_ groups in their PFCA backbones are defluorinated comparatively easily once decarboxylated, as the C—F bonds in the CF_2_ groups have relatively low BDEs (∼106 kcal/mol) [5]. In contrast, shorter PFCAs are more challenging to defluorinate because their –CF_2_ groups have stronger C—F bonds (>109 kcal/mol) [5]. **C**_**2**_ **F**_**5**_ **A** and **C**_**3**_ **F**_**7**_ **A** were determined to have the free F^–^ recovery rates of 36.2% and 33.7%, respectively, according to IC data for their post‐CCE solutions (Figure 4B), as expected for short‐chain PFCAs and in accordance with the rationale discussed above. We also determined that the **ACF**_**2**_ **A** substrate was degraded by 77% under identical CCE conditions (Figure S20) [30]; this higher degradation is ascribable to the absence of a terminal CF_3_ group in the structure of **ACF**_**2**_ **A**. However, **ACF**_**2**_ **A** exhibited a fluoride recovery rate of 43%, which is possibly due to fewer CF_2_ groups in its structure.

### 2.3 Defluorinating Fluorotelomers

**FIGURE 5:**
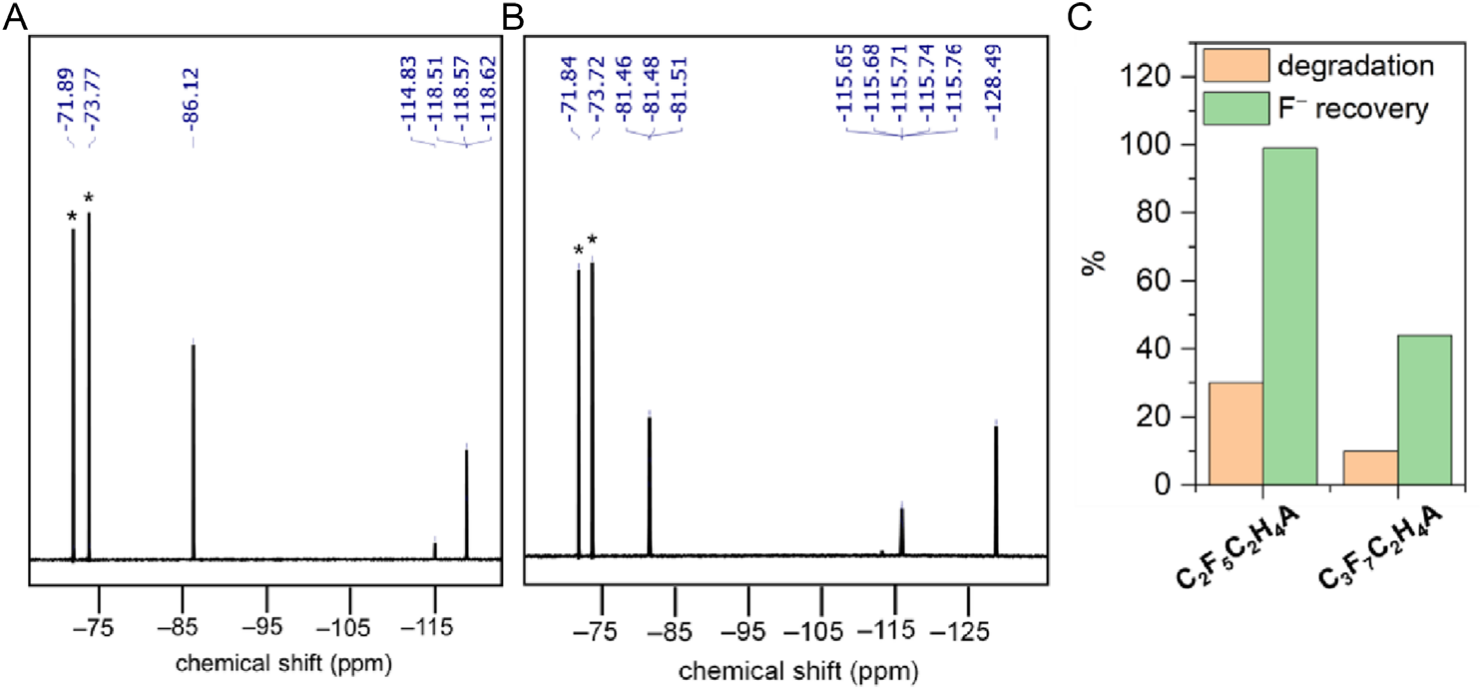
^19^F NMR for catholyte solution collected after performing CCE in the presence of (A) **C**_**2**_ **F**_**5**_ **C**_**2**_ **H**_**4**_ **A** (36 mM) and (B) **C**_**3**_ **F**_**7**_ **C**_**2**_ **H**_**4**_ **A** (36 mM) and **[CuT2]ClO**_**4**_ (1 mM) over 8 h at –1 mA. (C) Degradation and fluoride recovery rates recorded for **C**_**2**_ **F**_**5**_ **C**_**2**_ **H**_**4**_ **A** and **C**_**3**_ **F**_**7**_ **C**_**2**_ **H**_**4**_ **A** under the identical electrochemical conditions. TBAPF_6_ was used as the internal standard, and its NMR peaks are marked with asterisks (*). ^19^F NMR peak integration values are listed in Table S1.

We also investigated the degradation and defluorination of fluorotelomers containing –CH_2_ groups between their –CO_2_H and –CF_2_ groups, specifically **C**_**2**_ **F**_**5**_ **C**_**2**_ **H**_**4**_ **A** and **C**_**3**_ **F**_**7**_ **C**_**2**_ **H**_**4**_ **A**. These fluorotelomers are challenging to defluorinate according to C—F BDE calculations for similar fluorotelomers owing to their strong C—F bonds (>109 kcal/mol) [5]. **C**_**2**_ **F**_**5**_ **C**_**2**_ **H**_**4**_ **A** (Figure 5A) and **C**_**3**_ **F**_**7**_ **C**_**2**_ **H**_**4**_ **A** (Figure 5B) were degraded by 30% and 10%, respectively, under the CCE conditions used in this study (–1 mA for 8 h) in the presence of the **[CuT2]ClO**_**4**_ catalyst. Interestingly, **C**_**2**_ **F**_**5**_ **C**_**2**_ **H**_**4**_ **A** was defluorinated by 99%, which indicates that the fragmented **C**_**2**_ **F**_**5**_ **C**_**2**_ **H**_**4**_ **A** molecules were comprehensively mineralized into free F^–^ without producing any other side products. Furthermore, **C**_**3**_ **F**_**7**_ **C**_**2**_ **H**_**4**_ **A** was defluorinated by 44% under identical electrochemical conditions, despite being only degraded by 10% (Figure 5C). Taken together, these results suggest that **[CuT2]ClO**_**4**_ activates C—F bonds highly efficiently under homogeneous CCE conditions in the MeCN electrolyte.

### 2.4 Proposed Mechanism for the Electrochemical Activation of C—F Bonds

**FIGURE 6:**
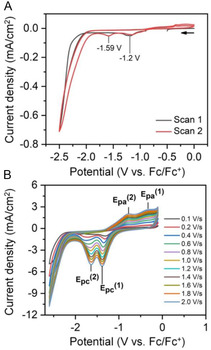
(A) CVs traces recorded for **[CuT2]ClO**_**4**_ in N_2_‐saturated 0.1 M TBAClO_4_ MeCN solution in the presence of 64.5 mM **TFE** upon repeating CV cycling. Scans 1 and 2 in the figure correspond to the first and second CV scans, respectively, at 0.1 V/s. (B) Second CV sweeps (Scan 2) for the electrolyte described in panel (A) at various scan rates in the 0.1–2.0 V/s range.

We recorded CV with **TFE** as the substrate to understand how resilient C—F bonds are activated using the homogeneous Cu(I) electrocatalyst because **TFE** contains only a single CF_3_ group as its fluorinated alkyl group, which enabled us to explore interactions between **[CuT2]ClO**_**4**_ and the CF_3_ group. The addition of **TFE** to the MeCN electrolyte in the presence of **[CuT2]ClO**_**4**_ resulted in current enhancements at potentials more negative than –2 V versus Fc/Fc^+^ (Figure 6). These catalytic currents continued to increase as the **TFE** concentration was further increased to 64.5 mM in the MeCN electrolyte (Figure S21), at which point the peak current densities began to saturate. The higher catalytic currents at potentials more negative than –2 V versus Fc/Fc^+^ are ascribable to proton (H^+^) reduction from **TFE** (p*K*_a_ = 35.4 in MeCN) [41], as confirmed by the H_2_ detected in the headspace of the catholyte via GC during CCE. However, more than 60% of the catalytic current observed is ascribable to the hydrogen‐evolution reaction (HER) in the presence of **[CuT2]ClO**_**4**_ and **TFE**, which overlapped with the background HER current associated with the bare glassy carbon electrode (GCE) in the absence of the Cu(I) catalyst at identical **TFE** concentrations (Figure S22).

The addition of a small amount of **TFE** (i.e., 9.2 mM) resulted in an increase in the peak current density at around –1.2 V versus Fc/Fc^+^, where ligand reduction occurs, accompanied by an additional cathodic wave at around –1 V versus Fc/Fc^+^. The peak near –1.2 V versus Fc/Fc^+^ shifted by ∼100 mV toward more positive potentials as the **TFE** concentration was increased from 9.3 to 64.5 mM under identical electrochemical conditions (Figure S21A). We analyzed the cathodic peak shift near –1.2 V versus Fc/Fc^+^ at various **TFE** concentrations in the MeCN electrolyte and found that the peak potential changed by 61 mV per logarithm of the TFE concentrations (Figure S21B), indicative of an electron‐transfer‐controlled event (theoretically, 58.2 mV/dec at 20°C) in the presence of **TFE** [42]. Identical electrochemical experiments performed in the absence of **[CuT2]ClO**_**4**_ did not exhibit such additional redox features. We hypothesize that the additional reductive waves at –1.2 V versus Fc/Fc^+^ are associated with **TFE** defluorination assisted by **[CuT2]ClO**_**4**_. This hypothesis was further evidenced by collecting CV traces for **[CuT2]ClO**_**4**_ using ethanol instead of **TFE** in the MeCN electrolyte, which did not reveal any such additional cathodic waves between –1 and –2 V versus Fc/Fc^+^, with the exception of the current enhancement ascribable to the HER at potentials more negative than –2 V versus Fc/Fc^+^ (Figure S23). The magnitude of the catalytic current observed for the HER promoted by **[CuT2]ClO**_**4**_ in the presence of ethanol is similar to that observed when an identical amount of **TFE** was used. **TFE** and ethanol are expected to use the HER active sites of the electrocatalyst, considering that ethanol and **TFE** are very weak acids (p*K*_a_ > 35) in MeCN. Therefore, the additional two reductive waves observed between –1 and –2 V versus Fc/Fc^+^ in the presence of **TFE** but not ethanol reveal that the CF_3_ group in **TFE** interacts with the active site in **[CuT2]ClO**_**4**_.

Successive CV sweeps recorded for **[CuT2]ClO**_**4**_ in the presence of **TFE** revealed an additional reduction wave at a potential (E_pc_^(2)^) of –1.59 V versus Fc/Fc^+^, along with the previously observed wave (E_pc_^(1)^) at –1.2 V versus Fc/Fc^+^ (Figure 6A). In addition, increasing the CV scan rate enhanced the reductive peak current densities at E_pc_^(1)^ and E_pc_^(2)^, and two oxidative redox waves were observed at E_pa_^(1)^ and E_pa_^(2)^ (Figure 6B) in the return CV sweep upon completion of the forward scan. We believe that the observed increases in peak current density at E_pc_^(1)^ and E_pc_^(2)^ are related to the event responsible for activating the C—F bond **TFE**.

**FIGURE 7:**
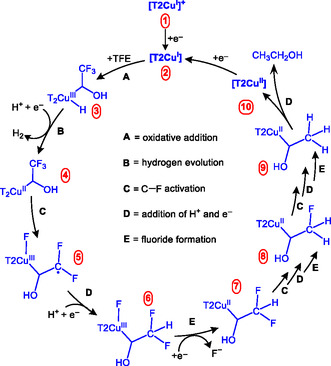
Proposed mechanism for the **[CuT2]** ^**+**^‐promoted electrochemical activation of the C—F bond in **TFE** in the MeCN electrolyte.

We propose a plausible mechanism for the **[CuT2]ClO**_**4**_‐promoted defluorination of **TFE** in a nonaqueous electrolyte based on the experimental evidence presented above (Figure 7). We assume that **[CuT2]ClO**_**4**_ remains ionized as **[T2Cu]** ^**+**^ (**1**) upon dissolution in the electrolyte, and that applying reductive conditions leads to the formation of **2**, in which the electron is delocalized over the triazole ligands [43]. This intermediate is then involved in C—F bond‐activation events with **TFE** during CCE under reductive conditions. **TFE** binds to the Cu center of **2** through oxidative addition to form organometallic intermediate **3**, which is similar to the intermediate observed during the **[T2Cu]** ^**+**^‐promoted activation of the C—Cl bond in CH_2_Cl_2_ reported previously by us [43]. H_2_ is generated from **3** via the transfer of another H^+^ and e^–^ to afford intermediate **4**, which activates a C—F bond in the CF_3_ group to form radical intermediate **5**. According to Savéant and coworkers, such a radical is most likely formed via the reductive cleavage of a C—F bond in a nonaqueous solvent [44]. When 1 equiv. of the 2,2,6,6‐tetramethylpiperidine‐1‐oxyl (TEMPO) radical scavenger was included, free F^–^ was not observed in the post‐CCE solution following CCE at –6 mA using **[CuT2]ClO**_**4**_ and **TFE** in the MeCN electrolyte, which indicates that radical **5** is essential to the overall transformation. We believe that **5** accepts another H^+^ and e^–^ to form intermediate **6**, which undergoes a first round of defluorination to form Cu^II^ species **7**. As post‐CCE analysis of catholyte solutions containing **TFE** as the starting substrate did not show any **DFE** or **MFE** as side‐products, we propose that **7** becomes involved in a subsequent round of C—F activation, followed by 1H^+^/1e^–^ addition and F^–^ elimination to yield Cu^II^ intermediate **8**, which completes this series of reactions and the full **TFE**‐defluorination cycle.

## 3 Conclusions

We investigated the ambient activation of resilient C—F bonds in fluoromethylalkyls using the **[CuT2]ClO**_**4**_ molecular electrocatalyst under CCE conditions, with a particular focus on short‐chain PFCAs formed as end products during the mineralization of environmentally persistent PFASs. Free F^–^ was recovered in high (99%) yield from **MFE**, whose C—F BDE is 113.1 kcal/mol. In addition, we defluorinated the CF_3_ groups in **TFE** and **TFA**, whose C—F BDEs exceed 118.5 kcal/mol. We also investigated the degradation and defluorination of short‐chain PFCAs containing only one or two ‐CF_2_‐ units in their backbones, as well as two fluorotelomers; these compounds are common PFAS contaminants in groundwater. Short‐chain PFCAs exhibited defluorination rates of between 35% and 40%, whereas a fluorotelomer containing only one CF_3_ and one CF_2_ group as its fluorinated alkyl units was defluorinated by up to 99%. The formation of F^–^ was detected using ^19^F NMR spectroscopy and IC after bulk electrolysis at a constant reductive current of −6 mA for 12 h. Overall, this study demonstrated the potential of defluorinating challenging fluoromethylalkyl substances using a molecular Cu(I) electrocatalyst in a nonaqueous electrolyte at room temperature. The homogeneous electrochemical activation of C—F bonds using first‐row transition‐metal molecular catalysts is rare; to the best of our knowledge, only one example has been reported [30]. The electrochemical activities demonstrated by **[CuT2]ClO**_**4**_ in this study are impressive and are expected to advance molecular electrocatalyst designs aimed at activating strong C—F bonds.

## 4 Experimental Section

### 4.1 Materials and General Methods

All the chemicals were purchased from commercially available sources: **TFE** (Acros Organics, 99.8%), **DFE** (Fisher Scientific, 95%), **MFE** (Aladdin, ≥95%), **CF**_**3**_ **A** (Acros Organics, 99.5%), **C**_**2**_ **F**_**5**_ **A** (Acros Organics, 97%), **C**_**3**_ **F**_**7**_ **A** (Thermo Scientific, 99%), **ACF**_**2**_ **A** (Synthonix, 97%), **C**_**2**_ **F**_**5**_ **C**_**2**_ **H**_**4**_ **A** (Thermo Scientific, 90%), **C**_**3**_ **F**_**7**_ **C**_**2**_ **H**_**4**_ **A** (Combi‐Blocks, 97%), **TBAF•3H**_**2**_ **O** (Thermo Scientific, 99%), **TBAClO**_**4**_ (Sigma Aldrich, >99%), and **TBAPF**_**6**_ (Sigma Aldrich, >99%). All chemicals were used as supplied, unless noted otherwise. Solvents were purified prior to use by passing them through a column of activated alumina using an MBraun solvent purification system.

### 4.2 Synthesis

Synthesis and detailed characterization data of **[CuT2]ClO**_**4**_ were published in our previous report [30].

### 4.3 CV

Electrochemical measurements were performed using a Bio‐Logic VSP potentiostat. CV was carried out using a conventional three‐electrode cell with a glassy carbon (GC, surface area = 0.07 cm^2^) working electrode, a nonaqueous Ag/0.01 M AgNO_3_ in MeCN reference electrode, and a Pt‐wire counter electrode. The GC electrode was prepared by polishing on a cloth polishing pad using 5‐μ aluminum oxide polishing slurry, followed by a thorough deionized water rinse [39]. Cyclic voltammograms (CVs) were recorded by dissolving **[CuT2]ClO**_**4**_ to 1 mM concentration with 0.1 M tetrabutylammonium perchlorate (TBAClO_4_) in dry MeCN, unless otherwise noted. Ferrocene was used as an external standard, and all potentials were reported with respect to the ferrocene/ferrocenium couple (Fc/Fc^+^). Herein, the potential values discussed in Fc/Fc^+^ can also be referenced to a standard hydrogen electrode (SHE) by adding +0.63 V, as reported by Pavlishchuk and Addison [45].

### 4.4 Controlled‐Current Electrolysis

CCE experiments were performed in a split H‐cell with a Selemion DSV anion‐exchange membrane between the working and counter cells to preserve the electrogenerated products (Figure S24). GDS 2050 carbon paper (3 cm × 0.5 cm, Fuel Cell Store) working electrode, Pt‐plate counter electrode, and nonaqueous Ag/AgNO_3_ (0.01 M) reference electrode in MeCN were used for CCE experiments. The working cell was sealed using an airtight custom Teflon cap with tubing to transfer any gaseous products in the headspace to the gas chromatograph for analysis. Leak tests were performed upon each assembly of the cell to ensure all gaseous products were carried to the gas chromatography.

### 4.5 Gas Chromatography (GC)

The headspace of the H‐cell was monitored using an SRI 8610C chromatograph equipped with FID and TCD detectors. During CCE, the gaseous products in the cathodic chamber were injected into the sample loop of an SRI gas chromatograph equipped with a multiple gas analyzer MG#5 with N_2_ gas flow of 20 sccm. The gas chromatograph was equipped with a 0.5 m Hayesep D column, a 2 m Molesieve column, and a TCD detector. N_2_ was used as the carrier gas within the system. The gas chromatograph was linearly calibrated with a mixture of calibration standards, CO, H_2_, CO_2_, and N_2_ in various ratios for both detectors (Figure S25).

### 4.6 Nuclear Magnetic Resonance (NMR) Spectroscopy

^1^H and ^19^F NMR spectra were recorded on a Bruker AV400 spectrometer (400 MHz). All ^13^C NMR spectra were recorded on a Bruker DMX500 spectrometer (500 MHz). Chemical shifts are reported in ppm and referenced to residual solvent (CD_3_CN) resonance peaks.

The pre‐ and postelectrolysis solutions were analyzed by ^19^F NMR by mixing 400 μL of electrolyte solution, 100 μL of NMR solvent (CD_3_CN), and 100 µL of 0.1 M of tetrabutylammonium hexafluorophosphate (TBAPF_6_) in acetonitrile as the internal standard. The net concentration of the internal standard in the NMR sample is 0.016 M.

### 4.7 Ion Chromatography

IC analysis was carried out on Metrohm equipped with Metrosep A Supp 5 ‐ 150/4.0 column attached with a Metrosep A Supp 5 Guard/4.0. 0.1 M H_2_SO_4_ was passed as suppressor regenerant with the standard aqueous eluent supplied from the Metrohm. Four standard solutions of tetrabutylammonium fluoride trihydrate in MeCN were prepared for the calibration of fluoride peaks (Figure S4). To measure the concentration of fluoride ions, the post‐CCE solution was diluted by mixing 0.5 mL of the post‐CCE solution in 1 mL of MeCN.

### 4.8 Calculations for %fluoride Recovery Based on IC Data

The %fluoride recovery was estimated using the following equation

(1) $$\%\mathrm{fluoride}\;\mathrm{recovery}=\frac{\left[F^{‐}\right]}{{\left[PFAS\right]}_{d}\times N_{C‐F}}\times 100$$

where [*F* ^*–*^] = The concentration of fluoride measured using IC chromatography data (mol/L), [*PFAS*]_*d*_ is the concentration of degraded PFAS (mol/L) calculated based on ^19^F NMR data, *N*_*C—F*_ = the total number of C—F bonds in the parent PFAS molecule.

### 4.9 Gas Chromatography‐Mass Spectrometry (GC–MS)

The headspace of the H‐cell and the post‐CCE solutions were also analyzed using Agilent Technologies 7890B GC system equipped with a thermal conductivity detector (TCD). Gas analytes were detected by passing through a HP‐Molesieve column (30 m in length, 0.32 mm in diameter, and 25 µm film). The temperature for the detector was set 50°C for the oven. Helium was used as the carrier gas with a flow at 15 mL/min. The gas sample (10 µL) from the headspace of the postelectrolysis cell was injected using an air‐tight syringe.

### 4.10 Density Functional Theory (DFT) Calculations

DFT calculations were carried out with Gaussian 09 (Revision E.01) [46]. Geometry optimizations were performed at the unrestricted ωB97X‐D^4^ level of theory in solution (acetonitrile, *e* = 35.688) using the solvation model based on density (SMD) approach [47]. The Def2‐SVP basis set was employed for all other atoms (denoted BS1) [48, 49]. Additional single‐point calculations were performed in solution on all optimized geometries using the Def2‐TZVPP basis set for all atoms (denoted BS2) [48, 49]. Stability analyses were performed alongside analytical frequency calculations on all stationary points to ensure that geometries correspond to local minima (all positive eigenvalues).

All computed free energies include zero‐point vibrational energy corrections, thermal corrections, and entropies calculated using standard statistical thermodynamic methods at 298.15 K and a 1 M standard state. The computed Gibbs free energies were corrected using Grimme's quasiharmonic approach for entropic contributions and Head‐Gordon's quasiharmonic approach for enthalpy [50, 51].

## Supporting Information

Additional supporting information can be found online in the Supporting Information section.

## Funding

This study was supported by National Science Foundation (CHE‐2347912).

## Conflicts of Interest

The authors declare no conflicts of interest.

## Supporting information

Supplementary Material

## Data Availability

The data that support the findings of this study are available from the corresponding author upon reasonable request.
