# Supplementary material for: Homogeneous Copper(I) Electrocatalyzed Degradation of Ultra‐Short and Long‐Chain Perfluoroalkyl Substances
Source: ChemSusChem. 2026 Apr 18;19(8):e202502358. doi: 10.1002/cssc.202502358 (PMC13091075; doi:10.1002/cssc.202502358)
Supplement: Supplementary file 1 — Supplementary Material [file CSSC-19-e202502358-s001.pdf]

Supporting Information for

Homogeneous Copper(I) Electrocatalyzed Degradation of Ultra-Short and Long-Chain PFAS

Soumalya Sinha,[†] Ashwin Chaturvedi,[†] Nabin Pandey,[†] Julien A. Panetier,[§,*] and Jianbing "Jimmy" Jiang[†,*]

[†]Department of Chemistry, University of Cincinnati, Cincinnati, OH 45221, USA

[§]Department of Chemistry, State University of New York, Binghamton, NY 13902, USA

*Corresponding authors: Emails: panetier@binghamton.edu, and jianbing.jiang@uc.edu

Table of Contents

Figure S1. E_{WE} vs time profile for **TFE** in the presence of Cu(I) catalyst S3

Figure S2. E_{WE} vs time profile for **TFE** without Cu(I) catalyst S3

Figure S3. Stacked ^{19}F NMR for free F^- identification in non-aqueous media S4

Figure S4. Ion chromatography (IC) for **TFE** after CCE S5

Figure S5. ^{1}H NMR for $TBAClO_4$ in MeCN S5

Figure S6. The images taken for the glass vials upon reacting with AgOTf S6

Table S1. List of ^{19}F NMR integration values for the terminal CF_3 group S6

Figure S7. CV for **[CuT2]**$^+$ at different scan rates S7

Figure S8. SEM image for the bare carbon paper S7

Figure S9. SEM image for the carbon paper after dipping into MeCN containing 1 mM **[CuT2]**$^+$ S8

Figure S10. SEM image for the carbon paper after performing CCE at –6 mA using **[CuT2]**$^+$ and **TFE** for 12 h S8

Figure S11. EDX data for the bare carbon paper S9

Figure S12. EDX data for the carbon paper after performing CCE S9

Figure S13. CPE for **[CuT2]**$^+$ at –2.1 V and –2.3 V in the presence of **TFE** S10

Figure S14. Images captured for the carbon paper working electrodes after completion of 12 h of CPE and CCE S10

Figure S15. SEM image for the carbon paper after performing CPE at –2.1 V using **[CuT2]**$^+$ and **TFE** for 12 h S11

Figure S16. EDX spectroscopy data for the carbon paper after performing CPE at –2.1 V using **[CuT2]**$^+$ and **TFE** for 12 h S11

Table S2. %fluoride recovery obtained at different **[CuT2]**ClO_4 concentrations S11

Figure S17. Comparative ^{19}F NMR spectra before and after CCE at –1 mA in the presence of **CF_3A** and **[CuT2]**$^+$ S12

Figure S18. ^{19}F NMR for post-CCE solution after CCE in the presence of **C_2F_5A** S13

Figure S19. ^{19}F NMR for post-CCE solution after CCE in the presence of **C_3F_7A** S13

Figure S20. ^{19}F NMR for post-CCE solution after CCE in the presence of **ACF_2A** S14

Figure S21. ^{19}F NMR for post-CCE solution after CCE in the presence of **$C_2F_5C_2H_4$A** S15

Figure S22. ^{19}F NMR for post-CCE solution after CCE in the presence of **$C_3F_7C_2H_4$A** S16

Figure S23. CVs of **[CuT2]**$^+$ in the absence and presence of **TFE** S16

Figure S24. CVs of bare glassy carbon electrode at different concentrations of **TFE** S17

Figure S25. CVs of **[CuT2]**$^+$ in the absence and presence of ethanol S17

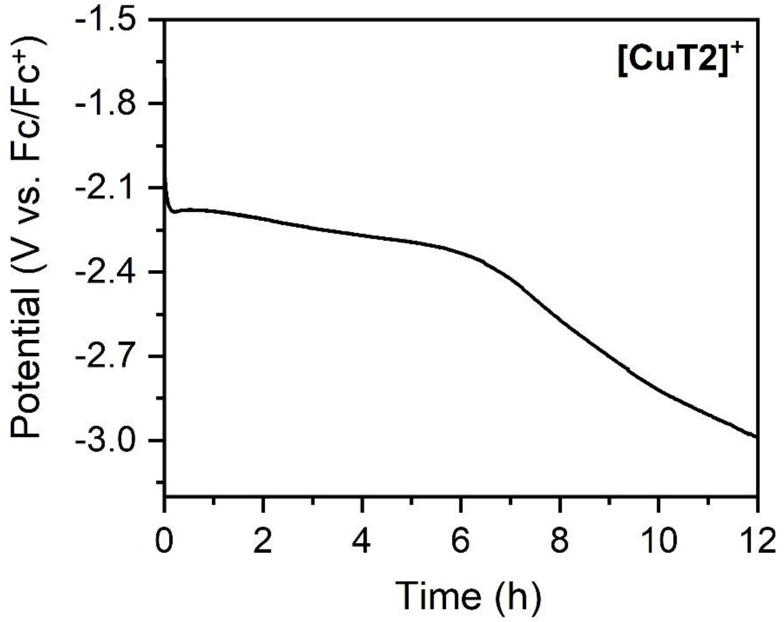

Figure S1. Change in potential over 12 h of controlled-current electrolysis performed at the constant current of –6 mA for **[CuT2]ClO4** (1 mM) in the presence of 0.86 M of **TFE** in N_2-saturated 0.1 M $TBAClO_4$ acetonitrile solution.

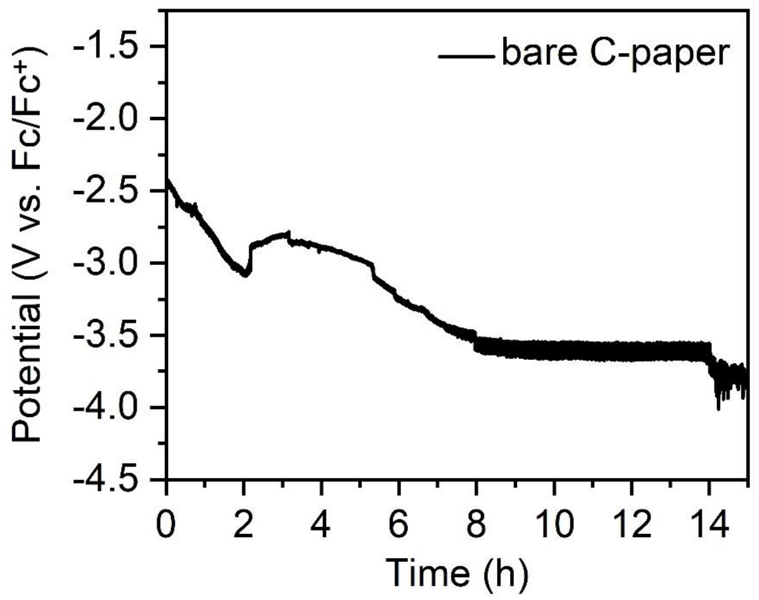

Figure S2. Change in potential over 15 h of controlled-current electrolysis performed at the constant current of –6 mA for bare carbon paper in the presence of 0.86 M of TFE in N_2-saturated 0.1 M $TBAClO_4$ acetonitrile solution.

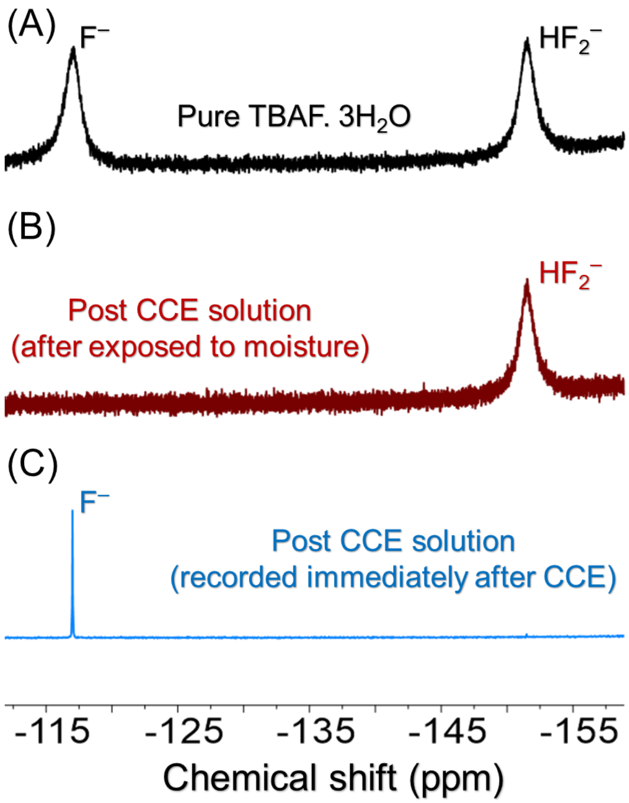

Figure S3. Stacked ^{19}F NMR (400 MHz) recorded for (A) pure tetrabutylammonium fluoride trihydrate (TBAF·3H_2O), (B) post-controlled-current electrolysis (CCE) catholyte solution after being exposed to air for 4 h, and (C) post-CCE catholyte recorded immediately upon the completion of CCE. CCE conditions: **[CuT2]ClO_4** (1 mM), **TFE** (0.86 M), –6 mA, 12 h, and 0.1 M $TBAClO_4$ in MeCN solution.

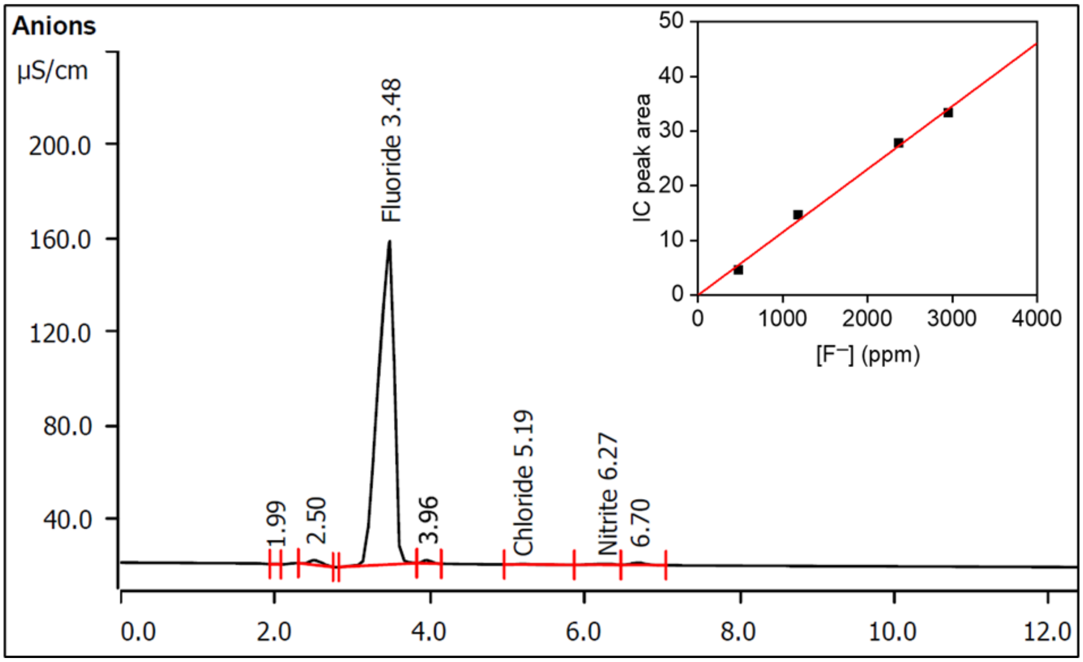

Figure S4. Ion chromatography (IC) data recorded for the post-electrolysis catholyte solution after performing 12 h of electrolysis at –6 mA of current in the presence of **[CuT2]ClO$_4$** and **TFE** in the N_2-saturated MeCN electrolyte. The IC calibration curve is shown in the inset.

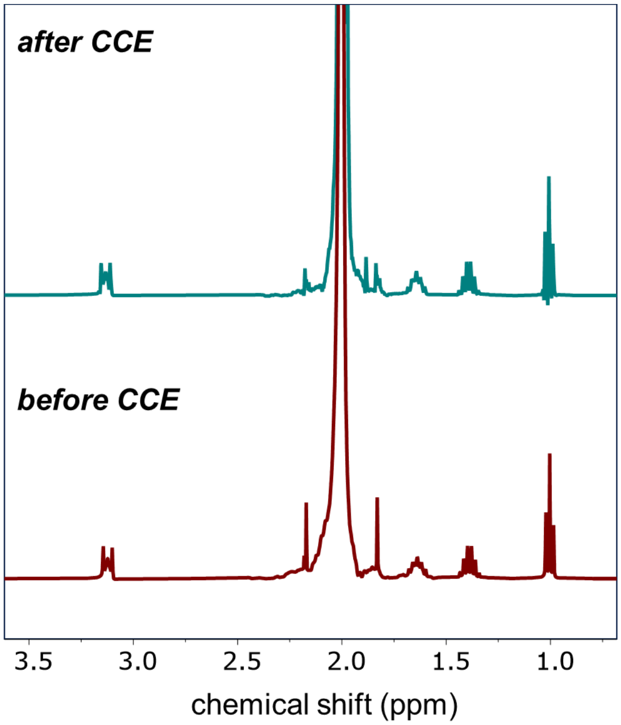

Figure S5. ^{1}H NMR recorded for 0.1 M tetrabutylammonium perchlorate (TBAClO$_4$) in MeCN before and after 12 h of CCE at –6 mA of cathodically applied current in the presence of **[CuT2]ClO$_4$** (1 mM).

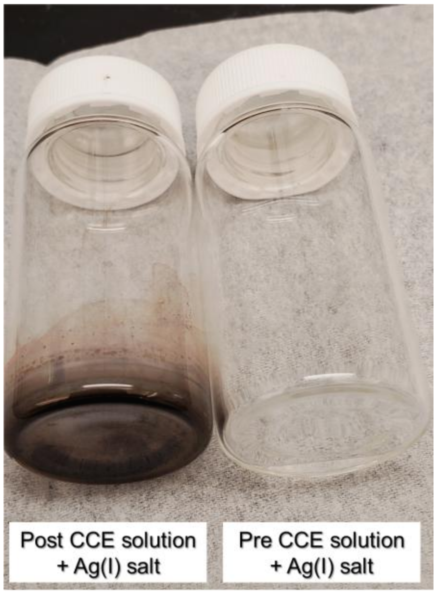

Figure S6. The images taken of the glass vials after the reaction between AgOTf and the catholyte solution, before (right) and after (left) performing controlled-current electrolysis (CCE), followed by filtration. The formation of the silver mirror on the glass vial was prominent when the post-CCE solution was used.

Table S1. List of ^{19}F NMR integration values for the terminal CF_3 group (highlighted in red) of the substrates studied in this report. For **DFE** and **MFE**, the terminal fluoroalkyl group has been considered.

substrate	before electrolysis	after electrolysis
CF_3-CH_2-OH (**TFE**)	117.68	8.78
HCF_2-CH_2-OH (**DFE**)	63.67	4.87
H_2CF-CH_2-OH (**MFE**)	31.37	2.68
CF_3-CO_2H (**CF_3A**)	6.66	2.17
CF_3-CF_2-CO_2H (**C_2F_5A**)	4.95	3.56
CF_3-CF_2-CF_2-CO_2H (**C_3F_7A**)	4.81	3.23
CO_2H-CF_2-CO_2H (**ACF_2A**)	10.35	2.38
CF_3-CF_2-CH_2-CH_2-CO_2H (**$C_2F_5C_2H_4A$**)	4.16	2.91
CF_3-CF_2-CF_2-CH_2-CH_2-CO_2H (**$C_3F_7C_2H_4A$**)	3.06	2.75

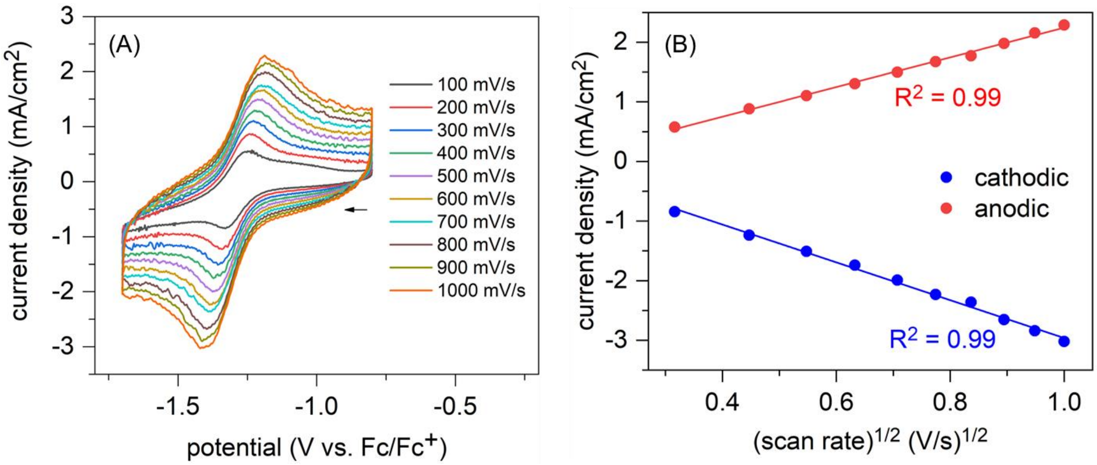

Figure S7. (A) Cyclic voltammograms recorded for **[CuT2]ClO$_4$** in N_2-saturated 0.1 M TBAClO$_4$ acetonitrile solution at different scan rates using a carbon paper working electrode (electrode area of 0.25 cm^2), a Pt plate counter electrode, and a nonaqueous Ag/AgNO$_3$ reference electrode. (B) The peak current densities observed at the cathodic and anodic waves were plotted with the square root of the scan rates.

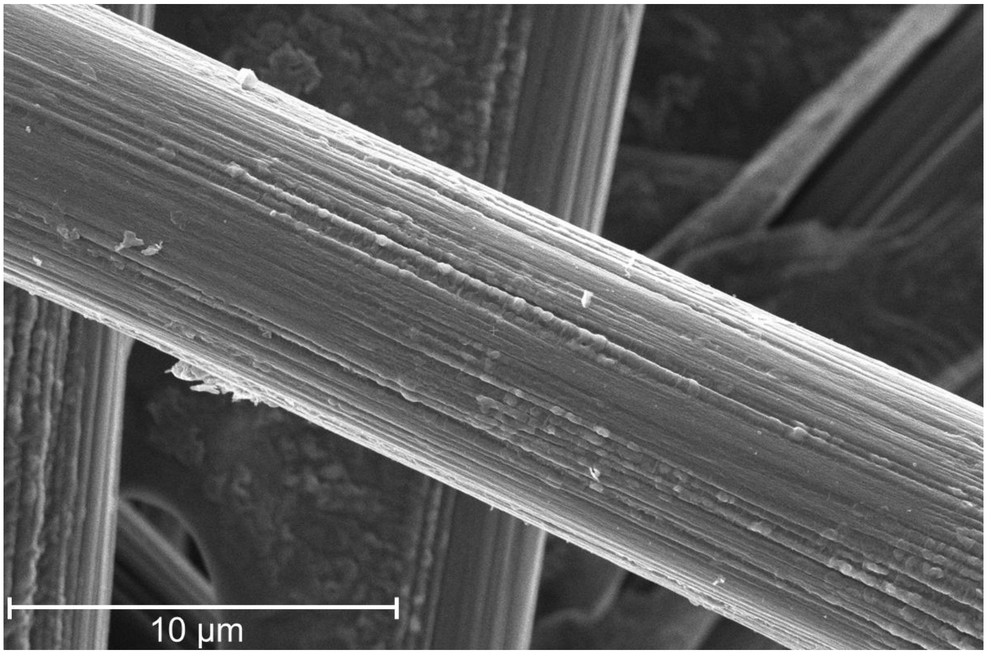

Figure S8. Scanning electron microscopy image for the bare carbon paper (GDS 2050).

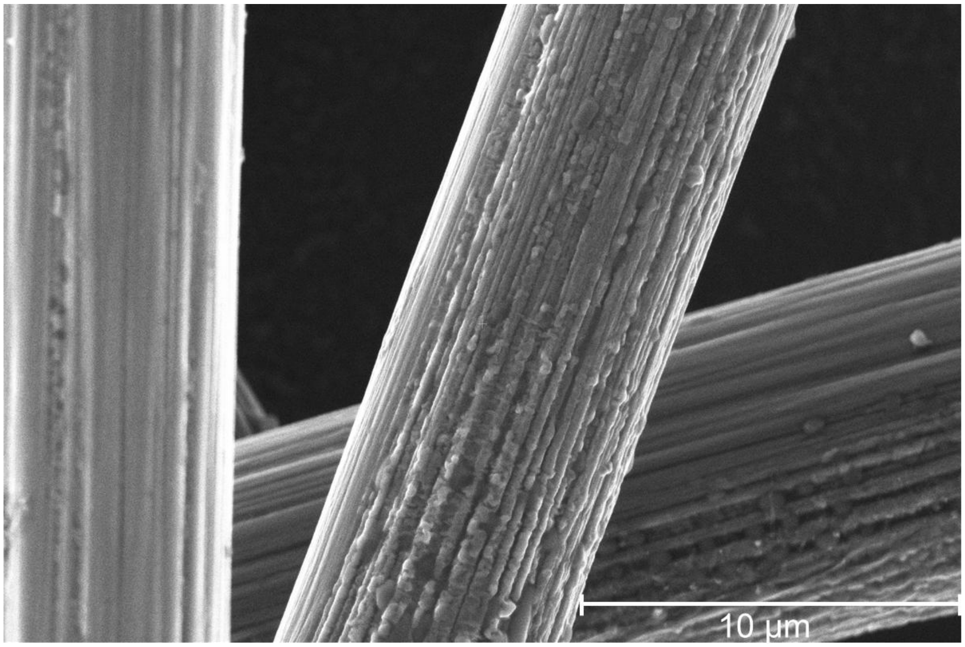

Figure S9. Scanning electron microscopy image for the carbon paper (GDS 2050) after dipping into an acetonitrile solution of 1 mM **[CuT2]ClO$_4$** for 15 mins, showing that barely any **[CuT2]ClO$_4$** complex got diffused into the carbon layers of the carbon paper.

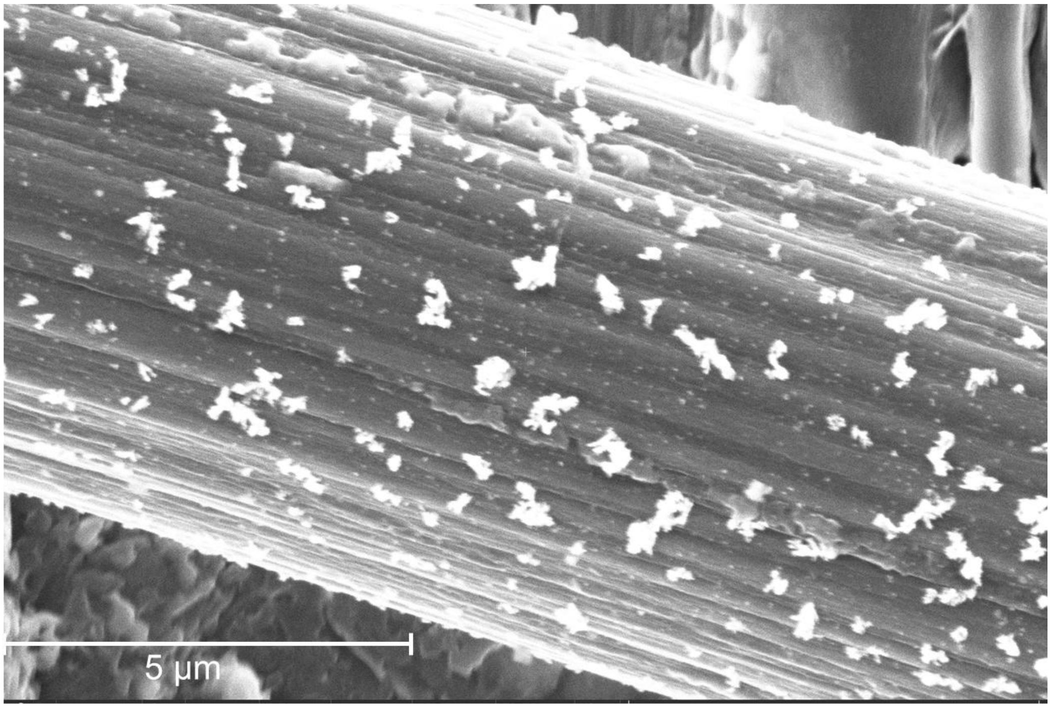

Figure S10. Scanning electron microscopy image for the carbon paper (GDS 2050) after performing controlled current electrolysis at –6 mA of current using 1 mM **[CuT2]ClO$_4$** and 0.86 M of **TFE** for 12 h. A small amount of metallic Cu deposition was observed.

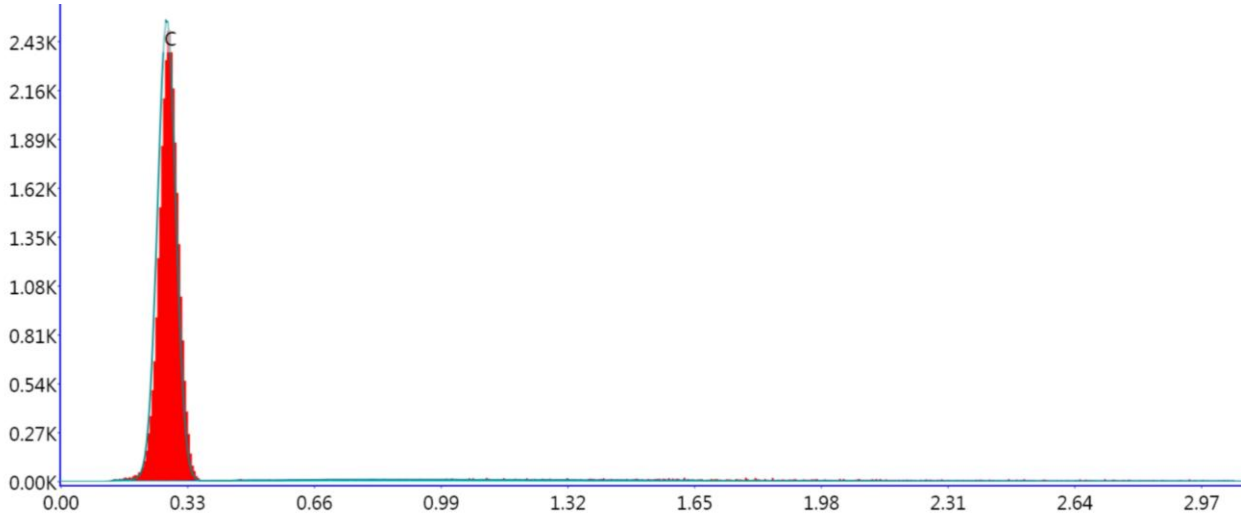

Figure S11. Energy dispersive X-ray spectroscopy data recorded for the bare carbon paper (GDS 2050) revealed the presence of carbon with an atomic percentage of 100%.

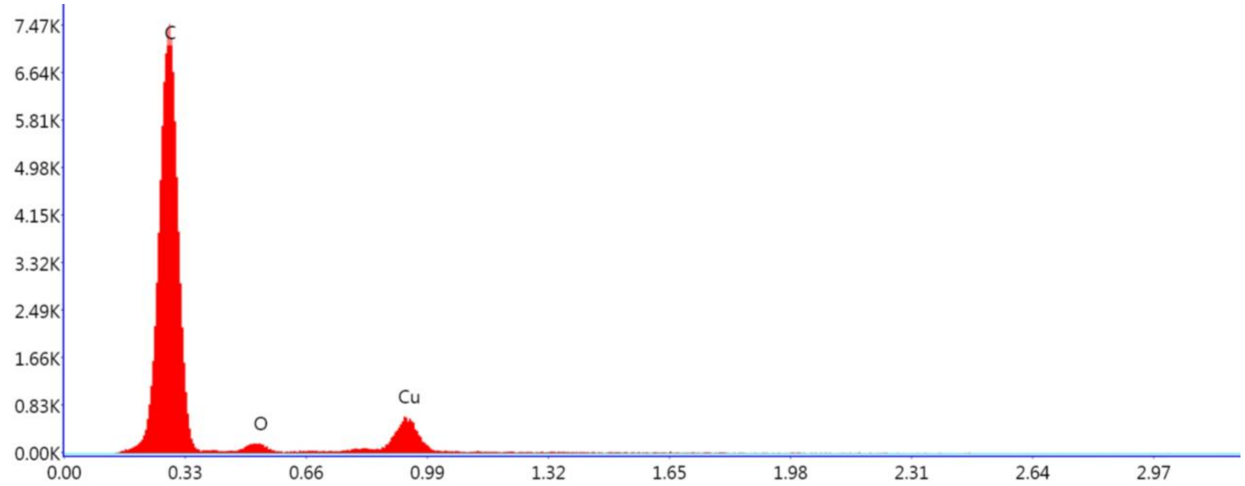

Figure S12. Energy dispersive X-ray spectroscopy data recorded for the carbon paper (GDS 2050) after performing controlled current electrolysis at 6 mA (reductive) of applied current using 1 mM **[CuT2]ClO$_4$** and 0.86 M of **TFE** for 12 h. The amount of deposited Cu was crudely estimated to be 2.47 atomic percent (C; 94.85%, O; 2.68%).

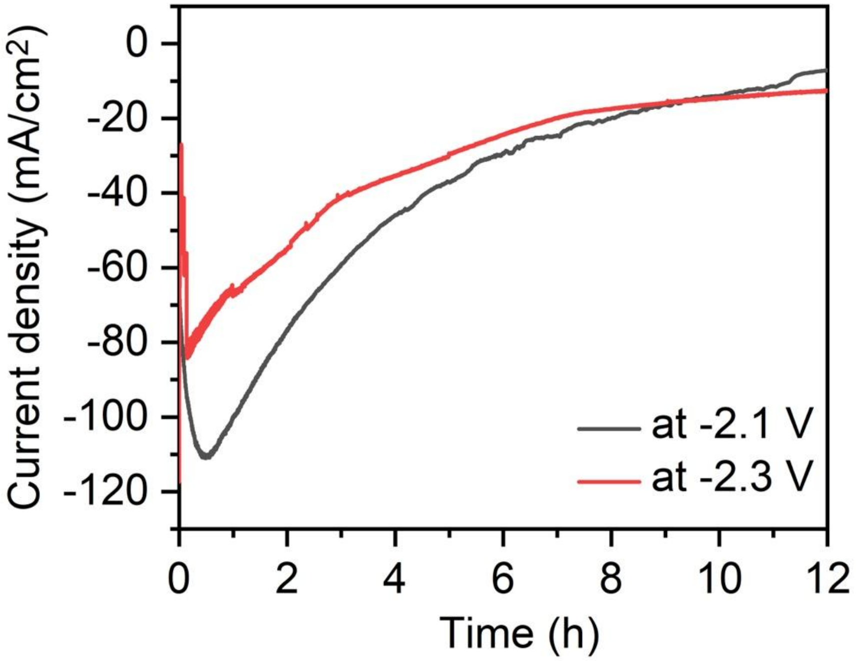

Figure S13. Controlled potential electrolysis for **[CuT2]ClO$_4$** (1 mM) at –2.1 V (black) and –2.3 V (red) vs. Fc/Fc$^+$ in the presence of 0.86 M of **TFE** in 0.1 M of TBAClO$_4$ MeCN solution.

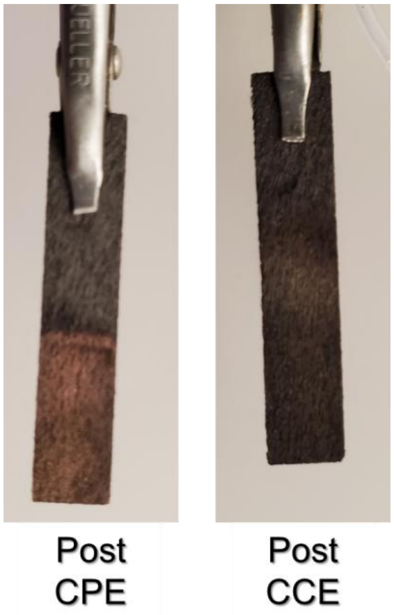

Figure S14. Images captured for the carbon paper working electrodes after completion of 12 h of controlled potential electrolysis (CPE, left) and controlled current electrolysis (CCE, right) for 1 mM of **[CuT2]ClO$_4$** in the presence of 0.86 M of **TFE** in the MeCN electrolyte. The potential and current applied for the CPE and CCE experiments are –2.1 V and –6 mA, respectively.

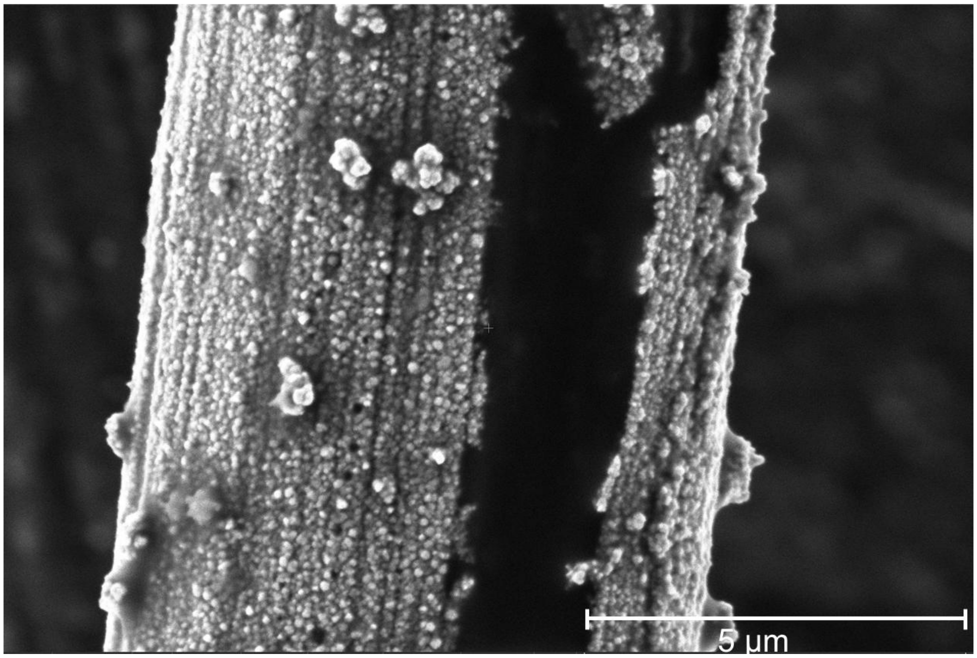

Figure S15. Scanning electron microscopy image for the carbon paper (GDS 2050) after performing controlled potential electrolysis at –2.1 V using 1 mM **[CuT2]ClO₄** and 0.86 M of **TFE** for 12 h. A layer of metallic Cu was deposited on the carbon surface.

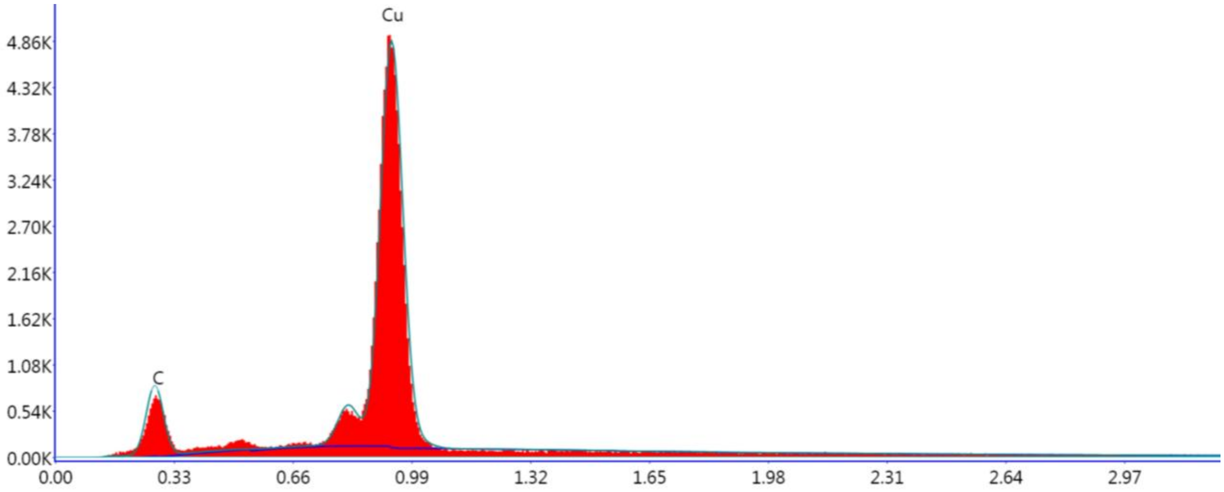

Figure S16. Energy dispersive X-ray spectroscopy data recorded for the carbon paper (GDS 2050) after performing controlled potential electrolysis at –2.1 V using 1 mM **[CuT2]ClO₄** and 0.86 M of **TFE** for 12 h. The amount of deposited Cu was crudely estimated to be 57.93 atomic percent (C; 40%).

Table S2. %fluoride recovery obtained at different **[CuT2]ClO4** concentrations.

**[CuT2]ClO4** concentration	%fluoride recovery	
	**DFE**	**MFE**
1 mM	19.2	99
5 mM	18.5	~100
10 mM	30.27	~100

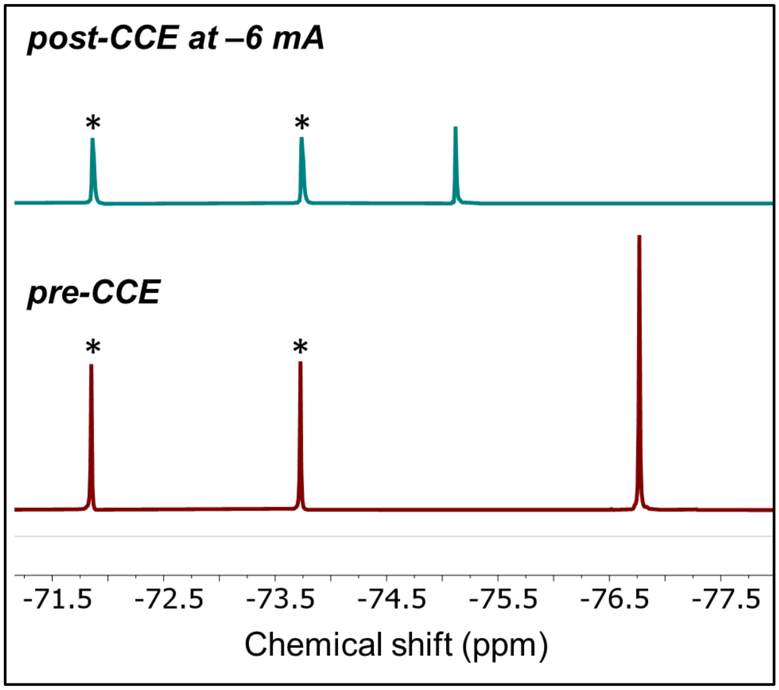

Figure S17. Comparative ^{19}F NMR spectra before and after performing controlled-current electrolysis at –6 mA in the presence of 36 mM **CF3A** and 1 mM **[CuT2]ClO4** in the MeCN electrolyte. ^{19}F NMR peak integration values are listed in Table S1.

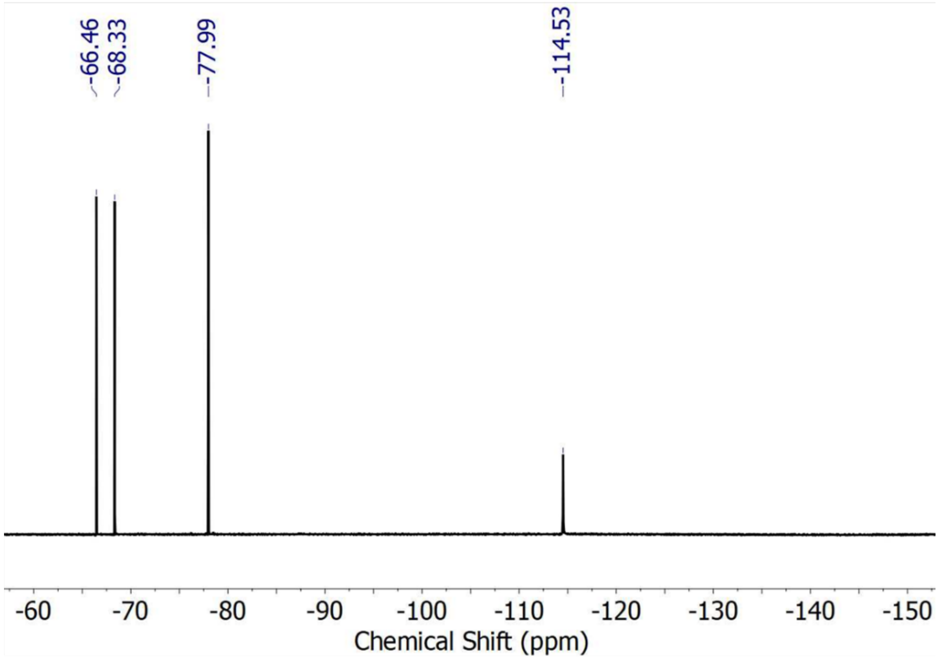

Figure S18. ^{19}F NMR for catholyte solution collected after performing controlled-current electrolysis in the presence of **C_2F_5A** (36 mM) and **[CuT2]ClO_4** (1 mM) over 8 h at –1 mA. ^{19}F NMR peak integration values are listed in Table S1.

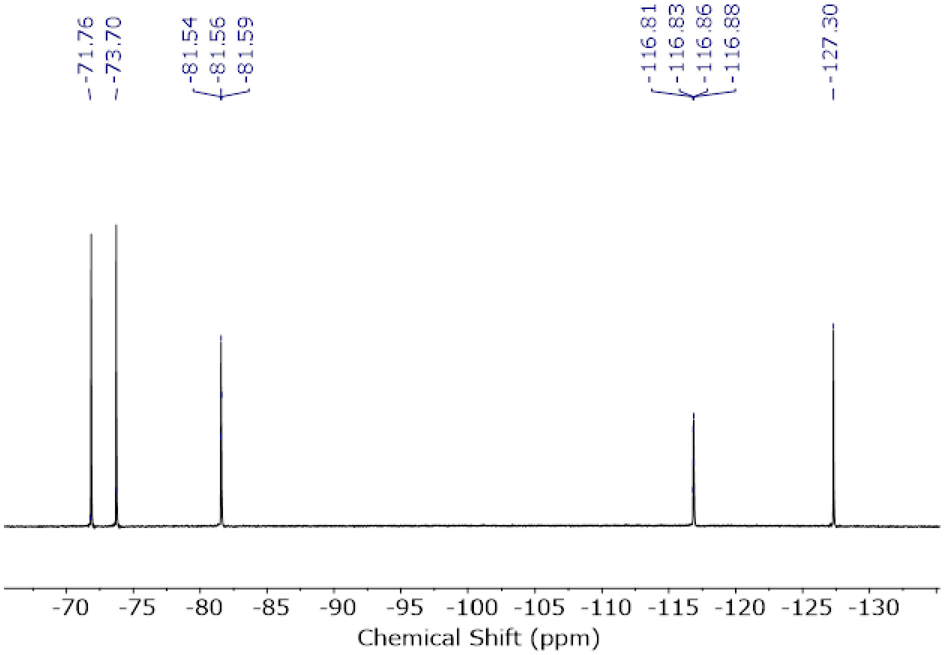

Figure S19. ^{19}F NMR for catholyte solution collected after performing controlled-current electrolysis in the presence of **C_3F_7A** (36 mM) and **[CuT2]ClO_4** (1 mM) over 8 h at –1 mA. ^{19}F NMR peak integration values are listed in Table S1.

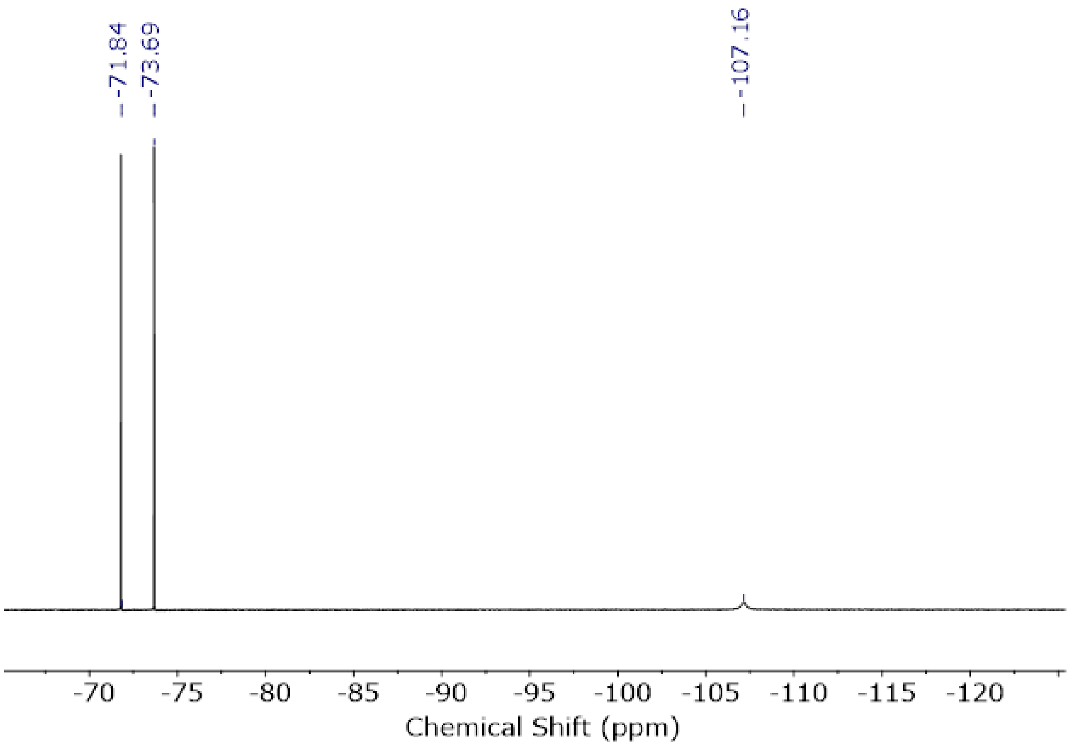

Figure S20. ^{19}F NMR for catholyte solution collected after performing controlled-current electrolysis in the presence of **ACF$_2$A** (36 mM) and **[CuT2]ClO$_4$** (1 mM) over 8 h at –1 mA. ^{19}F NMR peak integration values are listed in Table S1.

.

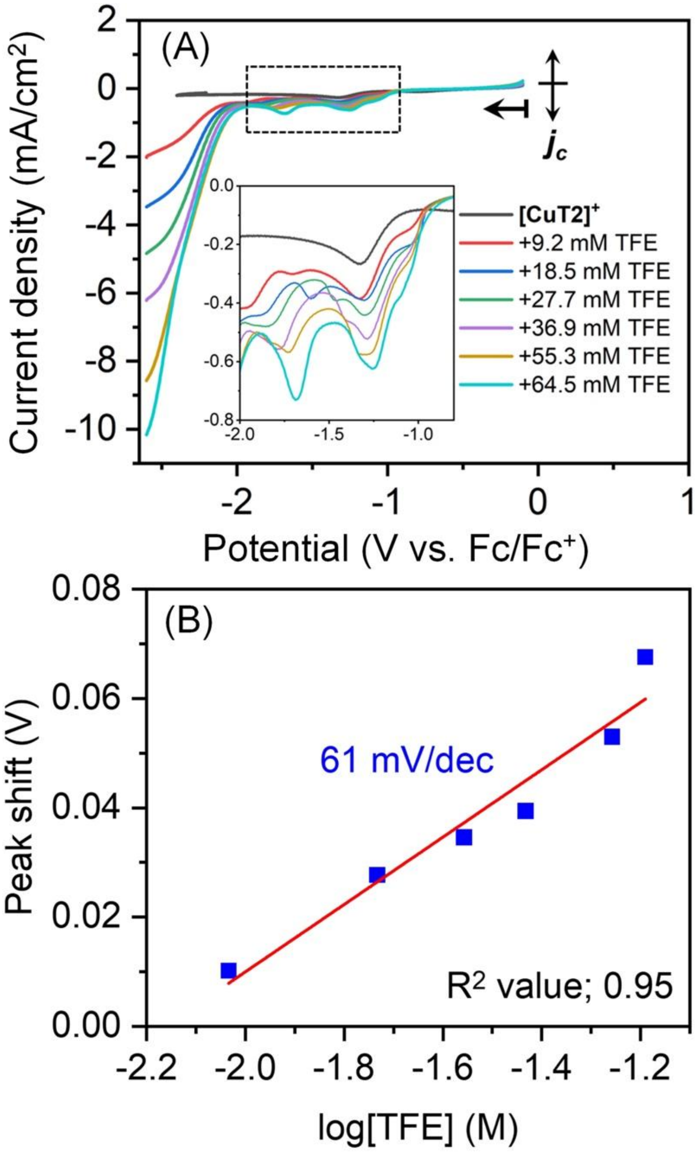

Figure S21. (A) Cyclic voltammograms recorded for **[CuT2]ClO$_4$** (1 mM) in the absence of **TFE** (black) and presence of **TFE** at different concentrations (9.2 mM – 64.5 mM) in N_2-saturated 0.1 M TBAClO$_4$ MeCN solution. Scan rate = 0.1 V/s. CVs within the potential window between –0.8 V and –2 V (dotted box) are highlighted in inset. (B) The peak shifts near –1.2 V were plotted with the logarithm of TFE concentrations as mentioned in (A).

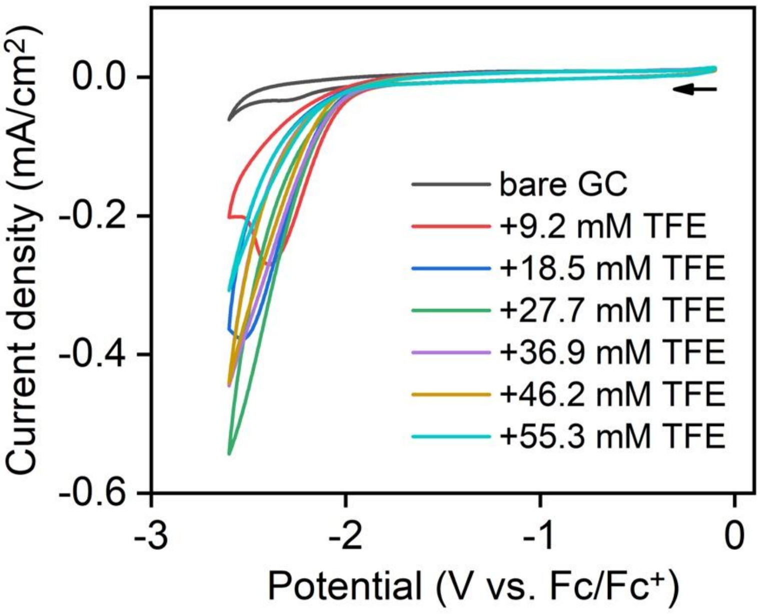

Figure S22. Cyclic voltammograms recorded for the bare glassy carbon electrode in N_2-saturated 0.1 M $TBAClO_4$ MeCN solution using different concentrations of **TFE** between 9.2 mM and 55.3 mM.

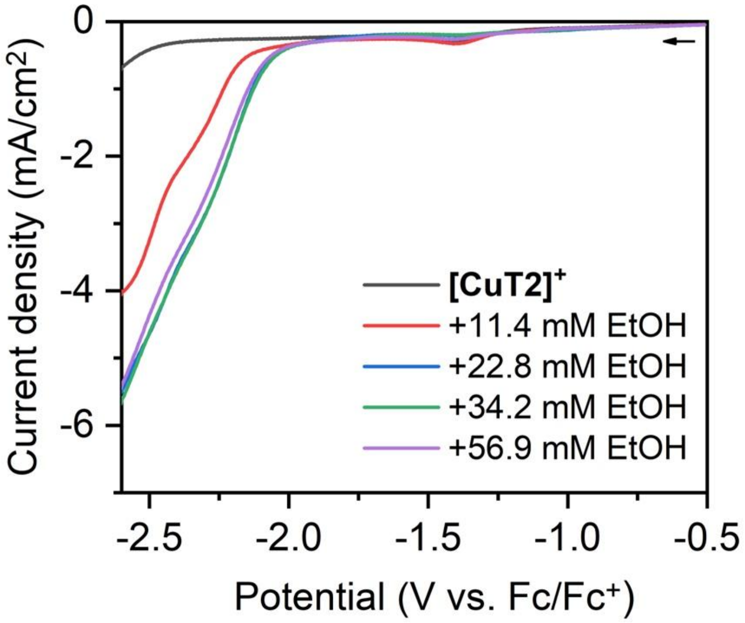

Figure S23. Cyclic voltammograms (CVs) recorded for **$[CuT2]ClO_4$** (1 mM) in the absence (black) and presence of ethanol at different concentrations, 11.4 mM (red), 22.8 mM (blue), 34.2 mM (green), and 56.9 mM (purple) in N_2-saturated MeCN electrolyte. Scan rate = 0.1 V/s. Note: successive CV sweeps did not show any additional redox features between –1.0 V and –2.0 V.

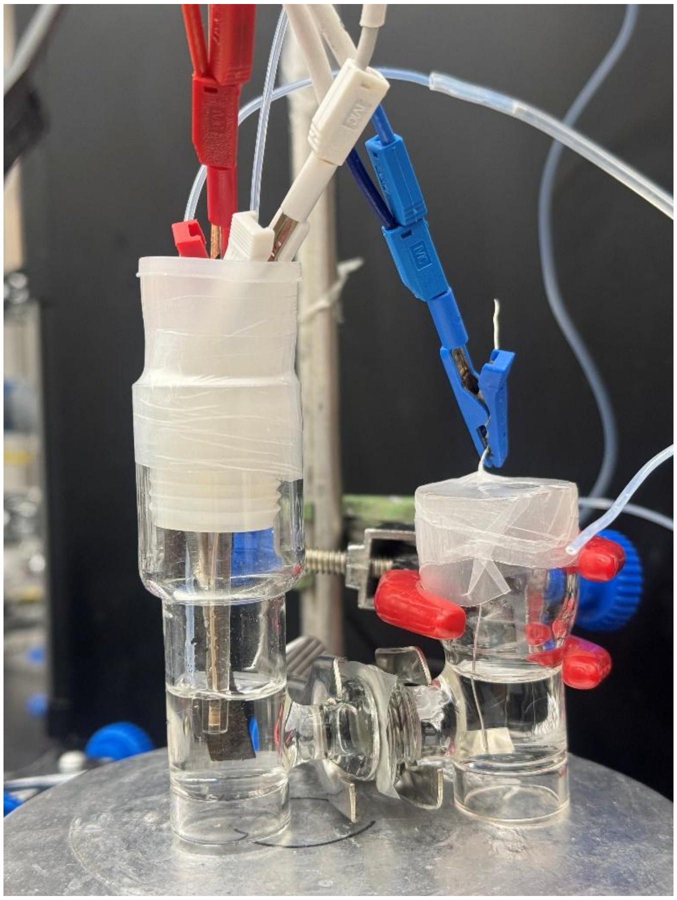

Figure S24. The picture of the electrochemical setup used for the controlled-current electrolysis experiments used in this report.

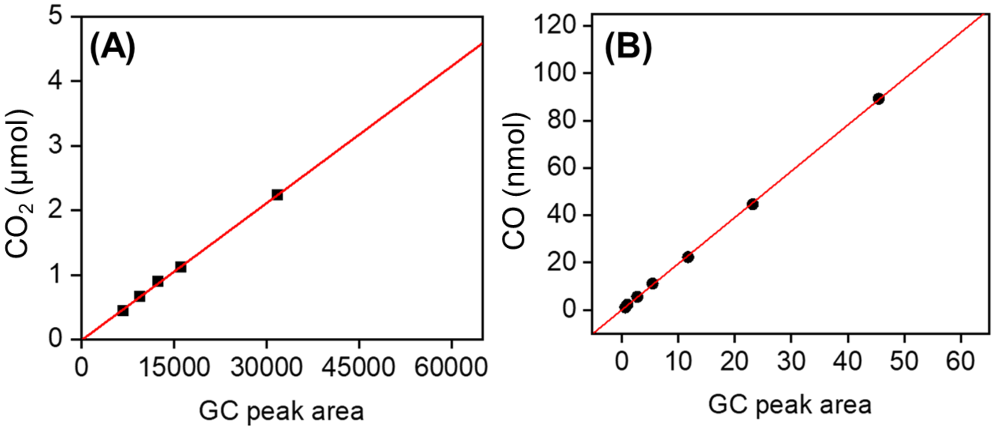

Figure S25. Sample GC calibration curves prepared using the standard gas mixtures of (A) CO_2 and (B) CO balanced with N_2.
